# A *Mycobacterium tuberculosis* fingerprint in human breath allows tuberculosis detection

**DOI:** 10.1038/s41467-022-35453-5

**Published:** 2022-12-14

**Authors:** Sergio Fabián Mosquera-Restrepo, Sophie Zuberogoïtia, Lucie Gouxette, Emilie Layre, Martine Gilleron, Alexandre Stella, David Rengel, Odile Burlet-Schiltz, Ana Cecilia Caro, Luis F. Garcia, César Segura, Carlos Alberto Peláez Jaramillo, Mauricio Rojas, Jérôme Nigou

**Affiliations:** 1 Cellular Immunology and Immunogenetics Group (GICIG), Institute of Medical Research, Faculty of Medicine, University Research Headquarters (SIU), University of Antioquia (UdeA), Medellin, Colombia; 2 Institute of Pharmacology and Structural Biology (IPBS), University of Toulouse, CNRS, University of Toulouse III-Paul Sabatier, Toulouse, France; 3 Interdisciplinary Group for Molecular Studies (GIEM), Institute of Chemistry, Faculty of Exact and Natural Sciences. University of Antioquia (UdeA), Medellin, Colombia; 4 Malaria Group, University Research Headquarters, University of Antioquia (UdeA), Medellín, Colombia; 5 Flow Cytometry Core, University Research Headquarters (SIU), University of Antioquia, UdeA, Medellín, Colombia

**Keywords:** Infectious-disease diagnostics, Lipidomics, Glycoconjugates

## Abstract

An estimated one-third of tuberculosis (TB) cases go undiagnosed or unreported. Sputum samples, widely used for TB diagnosis, are inefficient at detecting infection in children and paucibacillary patients. Indeed, developing point-of-care biomarker-based diagnostics that are not sputum-based is a major priority for the WHO. Here, in a proof-of-concept study, we tested whether pulmonary TB can be detected by analyzing patient exhaled breath condensate (EBC) samples. We find that the presence of *Mycobacterium tuberculosis* (Mtb)-specific lipids, lipoarabinomannan lipoglycan, and proteins in EBCs can efficiently differentiate baseline TB patients from controls. We used EBCs to track the longitudinal effects of antibiotic treatment in pediatric TB patients. In addition, Mtb lipoarabinomannan and lipids were structurally distinct in EBCs compared to ex vivo cultured bacteria, revealing specific metabolic and biochemical states of Mtb in the human lung. This provides essential information for the rational development or improvement of diagnostic antibodies, vaccines and therapeutic drugs. Our data collectively indicate that EBC analysis can potentially facilitate clinical diagnosis of TB across patient populations and monitor treatment efficacy. This affordable, rapid and non-invasive approach seems superior to sputum assays and has the potential to be implemented at point-of-care.

## Abstract (web summary)

Most conventional tuberculosis diagnostic tests rely on difficult to obtain sputum samples. In this proof-of-concept study, authors analyse whether pulmonary tuberculosis can be detected using exhaled breath condensate samples.

## Introduction

Tuberculosis (TB) remains one of the top ten causes of death worldwide and is the leading cause of death from a single infectious agent^1^. About a quarter of the world’s population is infected with *Mycobacterium tuberculosis* (Mtb), and thus at risk of developing TB. An estimated 10 million people developed TB in 2020 resulting in around 1.5 million TB deaths^1^. 11% of these TB cases occurred in children. An estimated one-third of all TB cases are, however, not diagnosed or reported, in part due to significant limitations in current diagnostic tools^1^. The WHO’s End TB Strategy aims for a 90% reduction in TB deaths and an 80% reduction in TB incidence by 2030 relative to 2015 levels. Diagnostic tools are urgently needed to both monitor progress and meet this goal^2^.

Three diagnostic priorities have been listed by the WHO and the TB community, including development of a point-of-care biomarker-based test for pulmonary TB^3,4^. Most conventional diagnostic tests rely on sputum samples, which can be difficult to obtain and have low diagnostic sensitivity in children, HIV-infected individuals and patients with extrapulmonary TB^3,5^. Therefore, the ideal diagnostic would not rely on sputum samples, and can also detect non-pulmonary TB. To be successfully implemented at point-of-care, a new test should use an easily accessible patient sample, such as urine, blood or breath condensate^3^. Blood-based diagnostics include interferon-γ release assays, which detect the host immune response to Mtb and have been relatively successful for monitoring latent TB. However, blood-based tests cannot accurately distinguish between Mtb infection and active TB disease^1^.

Since 2015, the WHO has recommended urine tests based on the detection of lipoarabinomannan (LAM), a mycobacterial cell envelope lipoglycan^6^ (Supplementary Fig. 1), to help diagnose TB in patients who are seriously ill with HIV^1,5,7^. However, urinary tests have suboptimal sensitivity, limiting their utility in TB screening^1^. One alternative is to test the liquid phase of exhaled air, called the exhaled breath condensate (EBC), which can be sampled by cooling^8–10^. Like urine and blood, the EBC is accessible and thus merits further investigation as a potential fluid that can be sampled for TB diagnosis^3^. Indeed, EBC collection is easy, relatively cheap, non-invasive and does not require specialized personnel. EBC reflects the composition of the airway lining fluid and may, therefore, contain lung disease-specific markers from infectious agents or infected host tissues^8–10^. Indeed, it was previously reported that fatty acid, oxidative stress and inflammatory mediator profiles in EBC can differentiate TB-infected adults and children from healthy controls^11,12^.

Here, we explored whether non-volatile bacterial molecules released into the extracellular milieu during infection can be detected in the EBC of TB patients. Interestingly, we found LAM at an unexpectedly high concentration range (15 to 120 µg/mL), as well as a set of Mtb-specific lipids and proteins in EBCs of TB patients. These factors were not detected in control individuals, either healthy or with community-acquired bacterial pneumonia. These Mtb-derived molecules allowed us to efficiently distinguish TB patients at baseline, including smear-negative and culture-negative adults. These markers also allowed us to distinguish samples from Mtb culture-positive or culture-negative children. Our data suggest that Mtb molecules in EBC are potential biomarkers for the early diagnosis of TB in adults and children, even in paucibacillary patients. Moreover, the longitudinal study of children under antibiotic treatment indicates that EBC analysis may allow real-time monitoring of treatment efficacy. Finally, we observed that LAM and lipids in EBC have a distinct structure compared to ex vivo cultured Mtb. This chemical difference indicates that bacilli in human lungs are using a distinct metabolic process compared to cultured Mtb. These data are consistent with Mtb growing as biofilms in the lungs and using host lipids as a major carbon source. Thus, EBC analysis gives previously elusive insights into the metabolic status of Mtb during lung infection and has the potential to diagnose TB patients.

## Results

We collected EBCs in health care centers in Medellín (Colombia) from adult and pediatric patients who had been diagnosed for pulmonary TB. Diagnosis was based on clinical, and/or radiographic, and/or bacteriological evidence of pulmonary disease resulting in treatment initiation by the diagnosing clinician^13–15^. Samples were collected using R-tubes^TM^ equipped with a 0.3-µm filter to avoid contamination with live bacteria. In 15 adult patients (Ad) out of 29, and in 5 pediatric patients (Ch) out of 17, the TB diagnosis was microbiologically confirmed by acid-fast bacilli smear (S^+^) and/or Mtb culture (C^+^) (Table 1). The first EBC sample was collected at baseline (prior to or within the first 2 weeks of initiating anti-TB treatment). Additional EBC samples were obtained for 6 pediatric patients at months 1 and 3 of anti-TB treatment (Table 1). Control individuals included 15 healthy adults, 15 healthy children, and 15 adult non-TB patients with community-acquired bacterial pneumonia (Table 1). Exclusion criteria were patients positive for HIV, diabetes, cancer, autoimmune diseases, immunosuppressive treatment, previous TB infections and smokers. These exclusions allowed us to focus on assessing TB in the absence of factors that influence disease or might confound detection. Subjects were asked to breathe at a normal frequency for 15 min, yielding at least 1 mL of condensate. For normalization between individuals, EBCs were lyophilized and resuspended in a total volume of 250 µL. Therefore, when available, the quantity of the bacterial molecules in EBC will be provided as mass per EBC.

**Table 1:** Clinical and demographic characteristics of the adult and pediatric TB patients, and control individuals, either healthy or with bacterial pneumonia

	Adult TB patients		
	Smear-positive	Smear-negative	
		Culture-negative	Culture-positive
Number of patients	8	7	14
Gender M/F	6/2	5/2	8/6
Age			
Median	34	44	38
Minimal	27	19	21
Maximal	62	70	68
TST^a^ (positive/performed)	7/8	5/7	8/14
TST diameter (mm)^b^	13 ± 2 (9–16)	11 ± 2 (3–14)	9 ± 5 (3–14)
BCG scar positive/total	6/8	6/7	10/14
Primary treatment	Isoniazid	Isoniazid	Isoniazid
	Rifampicin	Rifampicin	Rifampicin
Time of EBC collection	Under antibiotic treatment for <2 week	Before antibiotic treatment	Under antibiotic treatment for <2 week
Label	Ad S^+^	Ad S^-^C^-^	Ad S^-^C^+^

	Pediatric TB patients	
	String-test smear- or culture-positive	String-test smear- and culture-negative
Number of patients	5	12
Gender M/F	3/2	6/6
Age		
Median	9	9
Minimal	6	6
Maximal	12	12
TST^a^ (positive/performed)	3/5	11/12
TST diameter (mm)^b^	11 ± 5 (6–14)	9 ± 3 (4–16)
BCG scar (positive/total)	3/5	9/12
Primary treatment	Isoniazid	Isoniazid
	Rifampicin	Rifampicin
Time of EBC collection	Before antibiotic treatment	Under antibiotic treatment for <2 week, and at months 1 and 3^c^
Label	Ch S^+^/C^+^	Ch S^-^C^-^

	Control individuals			
	Community-acquired pneumonia		Healthy	
	Gram negative	Gram positive	Children	Adult
Number of persons	8	7	15	15
Gender M/F	4/4	4/3	10/5	12/3
Age				
Median	42	41	9	34
Minimal	18	20	7	26
Maximal	60	61	12	49
TST^a^ (positive/performed)	Unknown	Unknown	10/15	14/15
TST diameter (mm)^b^	Unknown	Unknown	0 ± 0.1 (0–0.3)	16 ± 5 (7–24)
BCG scar (positive/total)	Unknown	Unknown	10/15	14/15
Treatment	Moxifloxacin or Levofloxacin. Clindamycin	Moxifloxacin or Levofloxacin. Amikacin	N/A	N/A
Time of EBC collection	Before antibiotic treatment	Before antibiotic treatment	N/A	N/A
Label	Ad pneumo		Ch healthy	Ad healthy

*N/A* not applicable.

^a^Tuberculin skin test.

^b^Mean ± SD (range).

^c^Six (3M/3F; BCG scar positive; 6–12 years old) of the 12 patients were followed during antibiotic treatment and EBC collected after 1 and 3 months of antibiotic therapy.

### EBCs from TB patients contain high levels of LAM

**Fig. 1:**
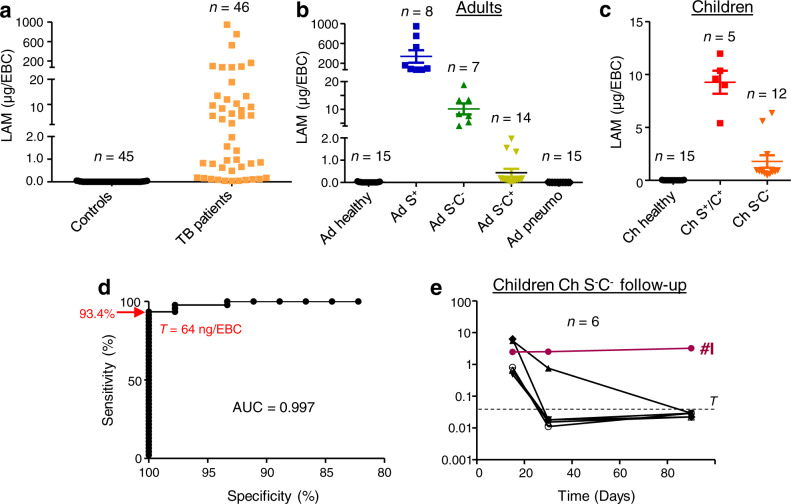
Quantification of LAM in EBC from TB patients and control individuals listed in Table 1. Quantity of LAM in EBC samples from all the subjects involved (**a**), adults (**b**), and children (**c**, **e**) was determined by an immunoassay using the CS-35 anti-LAM antibody. A receiver-operating characteristic (ROC) analysis of the LAM quantitation data is shown in **d**. AUC area under the curve, T threshold. In **a**, **b**, and **c**, the difference between TB patient groups and controls (healthy, pneumo) was statistically significant (Mann–Whitney *U*-test, two-tailed). Error bars represent SEM. Source data are provided as a Source Data file.

Since LAM is an established TB biomarker in urine^1,5,7,16^, we first attempted to detect LAM in EBCs using an anti-LAM antibody (CS-35^17^). Dot-blot analysis showed that EBCs of all TB patients at baseline exhibited a positive signal, whereas EBCs of control individuals, either healthy or infected by other pulmonary bacterial pathogens, showed a negative, or extremely weak, response (Supplementary Fig. 2). LAM content in EBCs was quantified relative to LAM purified from *M. tuberculosis* H37Rv using a standard curve (Supplementary Fig. 2). Unexpectedly, while the apparent concentration of LAM in urine from TB patients usually ranges from pg to ng/mL^5,7^, the apparent quantity of LAM in EBCs from TB patients at baseline ranged from 40 ng to 947 µg (Fig. 1a). More specifically the amount of LAM per EBC in different groups ranged from 70 to 947 µg in Ad S^+^, 4 to 19 µg in Ad S^-^C^-^, 0.04 to 1.5 µg in Ad S^-^C^+^, 5 to 12 µg in Ch S^+^/C^+^ and 0.48 to 6.4 µg in Ch S^-^C^-^ (Fig. 1b, c). In short, EBC samples from TB patients contain µgs of LAM (corresponding to concentrations in the µg/mL range), manifold higher than expected based on other patient fluids.

These unexpectedly high apparent amounts of LAM prompted us to validate whether LAM is indeed present in TB patient EBCs using cross-validation with a method other than the anti-LAM antibody. We therefore tested for the presence of pentose D-arabinose, a component of LAM^18^ (Supplementary Fig. 1). Pentose D-arabinose is present in mycobacteria and related genera, but is absent in eukaryotes^6^. After acid hydrolysis, chemical derivatization, and analyses by capillary electrophoresis monitored by laser-induced fluorescence (CE-LIF) and gas chromatography coupled to mass spectrometry (GC-MS), we could indeed detect arabinose in EBCs from TB patients but not control individuals (Supplementary Fig. 3a, b). More specifically, we detected D-, but not L-arabinose (Supplementary Fig. 3c). Interestingly, the monosaccharides mannose and glucose were also detected. Mannose is, in addition to arabinose, a major building block of LAM^6^; glucose is also a constituent of Mtb cell envelope compounds, such as the polysaccharide α-glucan or trehalose-based glycolipids. However, both mannose and glucose are also normally present in the human host. Arabinose was detected only after acid hydrolysis indicating that it is polymerized, whereas around 60% of glucose and 10% of mannose were found as free monosaccharides (Supplementary Fig. 3d; Supplementary Tables 1 and 2).

We then quantified arabinose using CE-LIF as a proxy for LAM in the EBC samples collected. LAM quantification by chemical analysis (Supplementary Fig. 4) yielded an overall pattern similar to that obtained for LAM quantification with the anti-LAM antibody (Fig. 1) and confirmed the µg abundance of LAM in EBCs from TB patients at baseline. However, chemical analysis provided numerical values distributed in a different range (0.7 to 30 µg vs. 10 ng to 947 µg by immunoassay).

We next examined the sensitivity and specificity of immunoassay-based LAM quantification in EBC for TB detection (Fig. 1a). These parameters were assessed using a receiver-operating characteristic (ROC) analysis (Fig. 1d). Overall ROC performance was assessed by calculating the area under the curve (AUC), which was 0.997 (95% CI, 0.9928 to 1.002; *n* = 46 cases, *n* = 45 controls; *P* < 0.0001). At a threshold of 64 ng/EBC, this ROC analysis yielded a sensitivity of 93.4% and a specificity of 100%. By these criteria, only 3 false negatives were present amongst 14 Ad S^-^C^+^ patients. However, it is worth noting that Ad S^-^C^+^ patients were under antibiotic treatment for 2 weeks or less (Table 1), and LAM content in EBC may have decreased compared to samples collected prior to treatment initiation, as suggested by the follow up study of pediatric patients (Fig. 1e). Leave-One-Out cross-validation yielded similar results, providing sensitivity, specificity and accuracy prediction values of 0.93, 0.98, and 0.96, respectively (Supplementary Fig. 5). Therefore, immunoassay-based LAM quantification in EBC allowed us to unambiguously distinguish most TB patients at baseline from healthy control subjects or non-TB patients with community-acquired bacterial pneumonia. This included detecting TB patients that were smear-negative and culture-negative adults (Ad S^-^C^-^) and smear- or culture-positive children (Ch S^+^/C^+^) (apparent LAM amount above 5 µg/EBC). The signal-to-noise ratio was thus found to be above 75. Even smear-negative and culture-negative pediatric patients (Ch S^-^C^-^) under antibiotic treatment for 15 days, with an apparent LAM content >480 ng/EBC, could be easily distinguished from control subjects (Fig. 1b, c).

Our longitudinal study followed 6 pediatric patients (Ch S^-^C^-^) for 3 months under antibiotic treatment. Antibody-based detection revealed a decline in apparent LAM content over the course of the treatment for 5 subjects (Fig. 1e). Four patients showed values below the threshold at month 1, and 5 at month 3. In contrast, apparent LAM content did not decrease over the time in EBCs collected from Child #I. It is worth noting that conventional antibiotic treatment (Table 1) for this patient failed. Child #I was subsequently referred to a search for primary immunodeficiency.

Altogether, these data suggest that LAM quantification by immunoassay in EBCs can be used to detect TB in adults and children, even in paucibacillary patients (smear-negative and/or culture-negative), and to monitor antibiotic treatment efficacy. These data indicate that diagnostics can potentially be based on EBC samples.

### LAM in EBCs has a non-mature polysaccharide structure

**Fig. 2:**
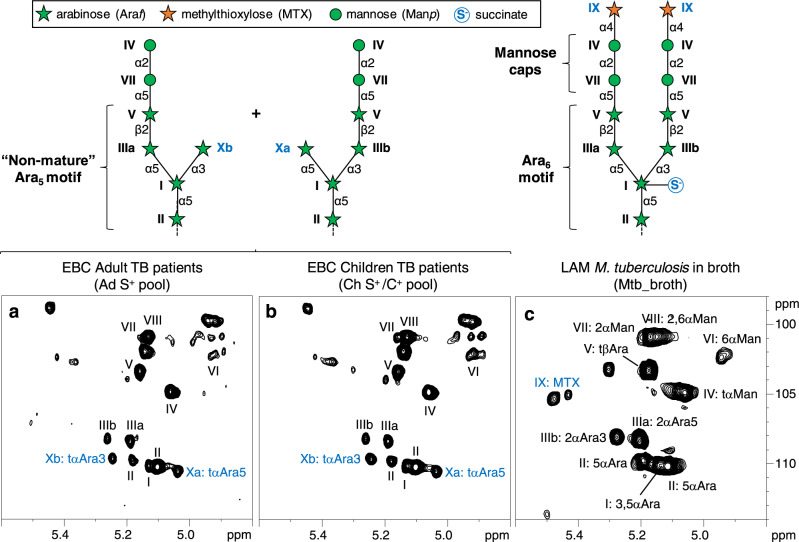
Characterization of LAM in EBC by NMR. Expanded region (δ ^1^H: 4.80-5.50, δ ^13^C 98-114) of the 2D ^1^H-^13^C HSQC spectrum in D_2_O at 298 K of Ad S^+^ pool (**a**) and Ch S^+^/C^+^ pool (**b**) LAM-enriched fractions, and Mtb_broth LAM (**c**). Cartoons show the structure of arabinan side chain termini deducted from NMR data. The branched hexa-arabinofuranoside (Ara_6_) motif is the main epitope of the CS-35 anti-LAM antibody. Structural motifs that differ between LAM in EBC and LAM purified from *M. tuberculosis* H37Rv grown in broth are highlighted in blue.

Quantification of LAM by immunoassay and chemical analysis showed a significant, but weak, correlation (*P* = 0.0002, *r* = 0.45; Supplementary Fig. 6). In addition, as mentioned above, numerical values obtained by each method were distributed in different ranges and were inconsistent in many cases. This disparity may be due to difficulties in accurate quantification using the CS-35 anti-LAM antibody and raises the question of the molecular structure of LAM released into human lungs. Having access to the structure of Mtb cell envelope compounds in vivo would give invaluable insight into the metabolic status of Mtb bacilli during human infection, a key step for the development of new chemo- and immuno-therapeutic or -diagnostic strategies^19^. We used two pooled EBC samples, one collected from 50 smear-positive adult TB patients (Ad S^+^ pool) and the other from 50 smear- or culture-positive pediatric TB patients (Ch S^+^/C^+^ pool) (Supplementary Table 1). The pooled samples should contain levels of LAM (several tens of micrograms) that are sufficient for structural analysis using a combination of highly sensitive analytical procedures we previously developed^20^. Ad S^+^ pool and Ch S^+^/C^+^ pool yielded 630 and 720 mg respectively of dried total material after freeze-drying (Supplementary Table 2). Lipids (around 1.5–2% w/w) were removed by organic solvent extraction, and the remaining material was submitted to enzymatic digestion to remove nucleic acids and proteins. The resulting LAM-enriched fractions contained 0.18 (Ad S^+^ pool) and 0.36 (Ch S^+^/C^+^ pool) mg of arabinose respectively (Supplementary Table 2). The fractions were analyzed by NMR using a 600 MHz spectrometer equipped with a cryogenic probe. The anomeric region of the 2D ^1^H-^13^C HSQC spectrum obtained with LAM-enriched fractions from both Ad S^+^ pool (Fig. 2a) and Ch S^+^/C^+^ pool (Fig. 2b) exhibited intense cross-peaks that could be unambiguously assigned to glycosidic units building LAM (Supplementary Fig. 1), i.e., 6-α-Man*p* (VI), 2-α-Man*p* (VII), 2,6-α-Man*p* (VIII), t-α-Man*p* (IV), 5-α-Ara*f* (II), 3,5-α-Ara*f* (I), t-β-Ara*f* (V), 2-α-Araf→5 (IIIa) and 2-α-Araf→3 (IIIb) residues, identical to that found in LAM purified from *M. tuberculosis* grown in broth (Mtb_broth) (Fig. 2c; Supplementary Table 3)^20–22^. This indicates that the LAM contained in EBCs from both adult and pediatric patients has an overall intact polysaccharidic structure, in agreement with the fact that LAM in EBCs is retained in dialysis tubing with a molecular weight cut off of 8-10 kDa. However, two additional cross-peaks were observed, previously assigned to t-α-Araf→5 (Xa) and t-α-Araf→3 (Xb) units evidenced in an EmbC mutant^22^. These residues typify a “non-mature” LAM, containing a reduced amount of branched hexa-arabinofuranosides (Ara_6_)^22,23^ and an unusual Ara_5_ motif (Fig. 2). Ara_6_ is the main epitope of the CS-35 anti-LAM antibody^17^. In LAM from slow-growing mycobacteria, including *M. tuberculosis*, some Ara_6_ chains are substituted at their non-reducing ends by mannose caps^6^ (Fig. 2 and Supplementary Fig. 1) that are key for LAM immunomodulatory properties^24^. 2-α-Man*p* are specific units of mannose caps. Corresponding signals (VII) were detected in NMR spectra of LAM-enriched fractions from both Ad S^+^ pool and Ch S^+^/C^+^ pool (Fig. 2 and Supplementary Table 3), albeit with a weaker intensity than in Mtb_broth LAM. This is consistent with the truncation of some of arabinan side chain termini that bear caps (Fig. 2). The presence of mannose caps on LAM in EBCs was confirmed by CE-LIF analysis after mild acid hydrolysis of LAM^25^. The electropherogram obtained with LAM-enriched fractions from both Ad S^+^ pool and Ch S^+^/C^+^ pool exhibited peaks corresponding to mono- (AM), α(1 → 2)-di- (AMM) and α(1 → 2)-tri- (AMMM) mannoside units (Supplementary Fig. 7a), as in Mtb_broth LAM^6^. Quantification^25^ indicated that there are approximately 1 mannosyl, 1.5 dimannosyl and 0.3 trimannosyl units per LAM molecule in both the Ad S^+^ and Ch S^+^/C^+^ pools, indicating a 3- to 4-fold reduced number of di- and tri-mannoside caps compared to that in Mtb_broth LAM^6^. LAM purified from Mtb_broth also contains minor covalent modifications on its polysaccharidic domain, such as methylthioxylose or succinyl motifs (Supplementary Fig. 1 and Fig. 2), whose biological significance remains largely unknown^6^. However, the signals corresponding to both motifs were absent in NMR spectra recorded for LAM-enriched fractions from both the Ad S^+^ and Ch S^+^/C^+^ pools (Fig. 2). Finally, the LAM polysaccharidic domain is attached to a mannosyl-phosphatidyl-*myo*-inositol (MPI) lipid anchor (Supplementary Fig. 1)^6^. Fatty acyl chains esterifying the MPI anchor allow LAM to migrate in sodium dodecyl-sulfate–polyacrylamide gel electrophoresis (SDS–PAGE)^26^. LAM-enriched fractions from Ad S^+^ pool and Ch S^+^/C^+^ pool were subjected to SDS–PAGE followed by western blot analysis. Bands corresponding to LAM were observed, indicating that LAM in EBCs likely contains an intact MPI anchor (Supplementary Fig. 7b). LAM in EBCs showed an apparent molecular weight that is slightly lower than that of Mtb_broth LAM, in agreement with a partial truncation of the arabinan side chain termini.

Altogether, detailed structural analyses indicate that LAM in EBCs from both adult and pediatric patients has an overall intact polysaccharide structure compared to LAM purified from *M. tuberculosis* H37Rv grown in broth. However, LAM from patients has significant modifications: (i) a reduction in branched Ara_6_ motifs with “non-mature” truncated arabinan side chain termini (Ara_5_), (ii) no MTX motif, and (iii) no succinylation detected. This analysis of LAM structure in patients is helpful for the rational development or improvement of diagnostic antibodies, or vaccines, targeting LAM^27^.

### Structure of *Mtb* lipids in TB patient EBCs typifies bacterial metabolism in human lungs

**Fig. 3:**
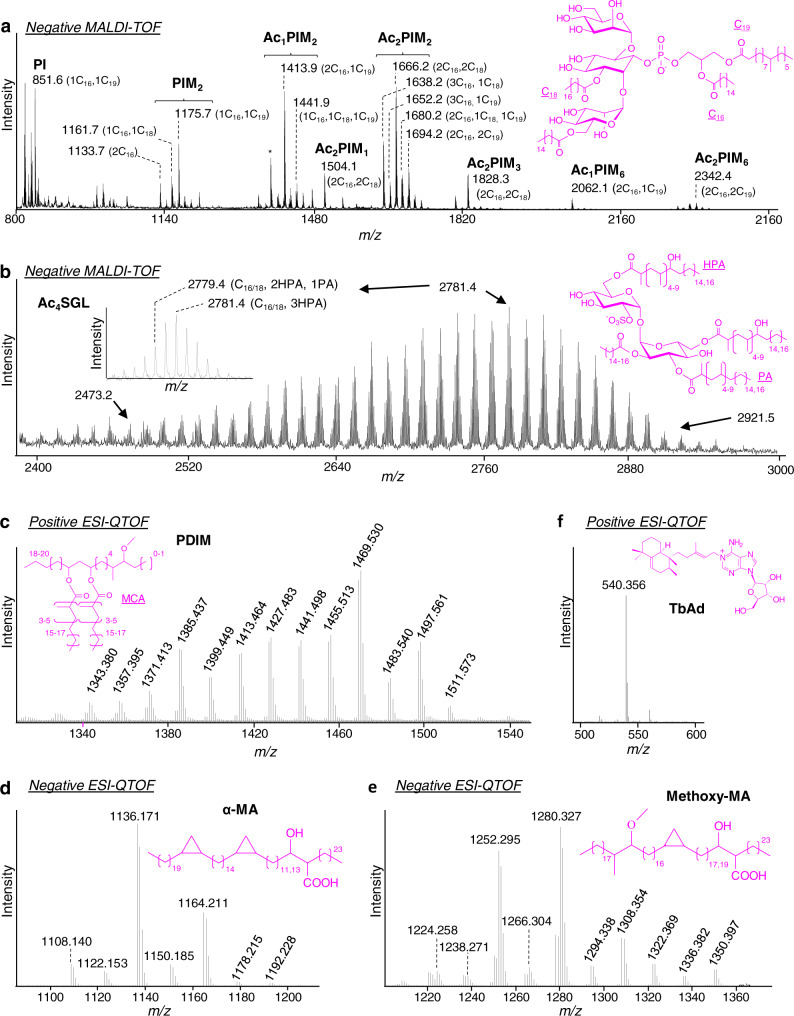
*M. tuberculosis* lipids and corresponding MS signatures detected in EBC from TB patients. **a** Negative MALDI-TOF mass spectrum of PI and PIMs. A structure of tetra-acylated PIM_2_ (Ac_2_PIM_2_) that contains 2 palmitic (C_16_), 1 stearic (C_18_) and 1 tuberculostearic (C_19_) acids is drawn. * indicate intense ions that do not correspond to PIM molecular species. **b** Negative MALDI-TOF mass spectrum of Ac_4_SGL. A structure that contains 2 hydroxyphthioceranyl (HPA) and 1 phthioceranyl (PA) (SL-II according to the nomenclature of Goren) is drawn. **c** Positive ESI-QTOF mass spectrum of PDIM. MCA, mycocerosic acid. **d**, **e** Negative ESI-QTOF mass spectrum of α- (**d**) and methoxy-(**e**) mycolic acids. The main forms are illustrated. **f** Positive ESI-QTOF mass spectrum of TbAd. 1-TbAd isomer is shown. Data are representative of at least 2 independent experiments on each EBC pooled sample. The precise stereochemistry of PIMs, SGLs, PDIM and Mycolic acids can be found in Minnikin & Brennan, 2020^28^. A detailed peak assignment is shown in Supplementary Tables 4–7.

*M. tuberculosis* produces highly abundant and structurally diverse lipids, some of which are species-specific^28^. As shown above, EBCs contain 1.5–2% (w/w) lipids (Supplementary Table 2). We tested whether *M. tuberculosis* lipids can be detected using mass spectrometry on pooled EBC lipid extracts. Five families of lipids, namely phosphatidylinositol mannosides (PIMs), sulfoglycolipids (SGLs), phthiocerol dimycocerosate (PDIM), mycolic acids (MA) and tuberculosinyladenosine (TbAd) were detected by either matrix-assisted laser desorption ionization-time of flight mass spectrometry (MALDI-TOF) and/or SFC-HRMS (supercritical fluid chromatography-high-resolution mass spectrometry) in both Ad S^+^ and Ch S^+^/C^+^ pooled samples (Fig. 3). As in Mtb_broth, PIM molecular species were present in two glyco-forms, Phosphatidylinositol di- (PIM_2_) and hexa- (PIM_6_) mannosides, existing in 3 main acyl-forms esterified by 2 (PIM_x_) to 4 (Ac_2_PIM_x_) fatty acyl chains (Fig. 3a)^29,30^. However, the acyl-form profile was more complex in EBCs, with a high abundance of unusual forms containing stearic acid (Fig. 3a and Supplementary Table 4). The PIM biosynthetic precursor, phosphatidylinositol (PI), was also detected (Fig. 3a). Negative MALDI mass spectrum also revealed a large distribution of tetra-acylated forms of SGLs (Ac_4_SGLs) as observed in Mtb_broth^31,32^, but with a shift towards higher molecular masses (Fig. 3b and Supplementary Table 5). This higher mass represented a mean extension of 7-carbon atoms per each of the 3 (hydroxy)phthioceranyl chains present on the molecule. The PDIM acyl-form profile was also modified, showing an increased proportion of the molecular species composed of longer alkyl chains (Fig. 3c and Supplementary Table 6). This likely represents a 3-carbon atom extension in each of the two mycocerosic acids that compose the molecule^33^. Interestingly, similar increases of Ac_4_SGLs and PDIM molecular masses have been previously reported for Mtb grown in vitro with propionate or cholesterol as the limiting carbon source, or in lungs of infected mice^33,34^. Methoxy- and α-mycolic acids were detected in their free fatty acid form (Fig. 3d, e and Supplementary Table 7). The distribution of the molecular species was very similar to that observed for mycolic acid esters in Mtb_broth^28,35^, the most abundant species being α-mycolic acids containing 78- or 80-carbon atoms (*m/z* at 1136.171 and 1164.211 respectively; Fig. 3d) and methoxy-mycolic acids containing 85- or 87-carbon atoms (*m/z* at 1252.295 and 1280.327 respectively; Fig. 3e). Notably, free mycolic acids are only present in trace amounts in planktonically grown cells of *M. tuberculosis*, in contrast to biofilm cultures where they are more abundant^36^. Finally, TbAd was detected in both isomeric forms (1-TbAd and *N*^6^-TbAd) at *m/z* 540.356^37^ (Fig. 3f). We then analyzed individual EBCs and observed that, when detected, the different *M. tuberculosis* lipids showed a profile of molecular species similar to that found in pools. This finding was consistent across TB patients, whatever their clinical and demographic characteristics. This lipid profile thus typifies the in vivo metabolism of bacilli in human lungs, suggesting bacteria are growing as a biofilm with host lipids as the carbon source.

**Fig. 4:**
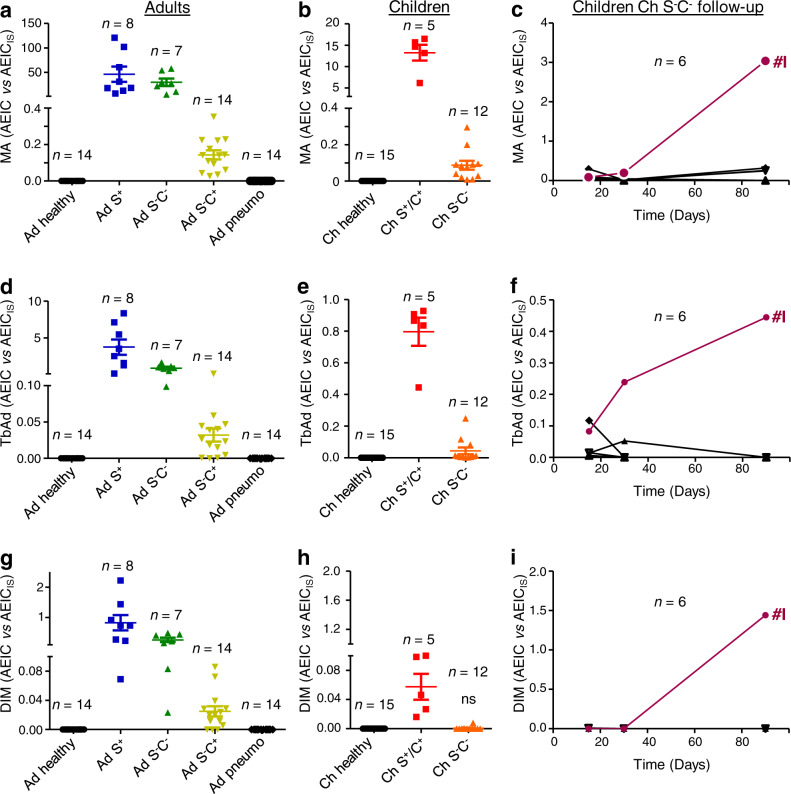
Abundance of *M. tuberculosis* lipids in EBC from TB patients and control individuals listed in Table 1. Abundance of MA (**a**–**c**), TbAd (**d**–**f**), and PDIM (**g**–**i**) per EBC from adults and children was determined by SFC-HRMS relatively to 1,2-ditridecanoyl-*sn*-glycero-3-phosphocholine (133 ng/mL of EBC) used as an internal standard (IS). Values are given as the ratio between areas of the extracted ion chromatograms (AEIC) of the ionized lipid molecular species and AEIC of the IS. In **g**, **h**, and **i**, values are multiplied by 10. In **a**, **b**, **d**, **e**, **g**, **h**, unless otherwise stated (ns, not significant), the difference between TB patient groups and controls (healthy, pneumo) was statistically significant (Mann–Whitney *U*-test, two-tailed). Error bars represent SEM. Source data are provided as a Source Data file.

In order to further characterize Mtb lipids as potential biomarkers, we performed a comparative analysis of the relative abundance of MA, PDIM and TbAd in the different samples based on mass spectrometry signal intensities (relatively to the signal of the 1,2-ditridecanoyl-*sn*-glycero-3-phosphocholine used as internal standard) (Fig. 4). The relative proportions of methoxy- vs. α-mycolic acids were constant across samples. Therefore, Fig. 4a–c show the cumulative relative abundances of both mycolic acid types. The overall distribution of MA, PDIM and TbAd was very similar to that obtained for LAM quantification (Fig. 1). The best correlation with LAM was obtained for MA (*r* = 0.54, *P* < 0.0001; Supplementary Fig. 6). Measurements of MA and TbAd showed a very good correlation (*r* = 0.90, *P* < 0.0001; Supplementary Fig. 6). The sensitivity of detection was higher for MA and TbAd than for PDIM. Indeed, as shown in Fig. 4h, i, we did not detect PDIM for smear-negative and culture-negative children (Ch S^-^C^-^), in contrast to LAM (Fig. 1c, e), MA (Fig. 4b, c) and TbAd (Fig. 4e, f). However, we only used 6% of the EBC for lipidomic analyses; there is therefore at least a 15-fold possible improvement in sensitivity. As shown for LAM, measurement of Mtb lipids in EBC may be used to diagnose TB in adult and pediatric patients, and to monitor antibiotic treatment efficacy. In contrast to LAM, which is shared by other mycobacterial species, some of these lipids are Mtb-specific.

### EBCs from TB patients contain *M. tuberculosis* proteins found in extracellular vesicles

**Fig. 5:**
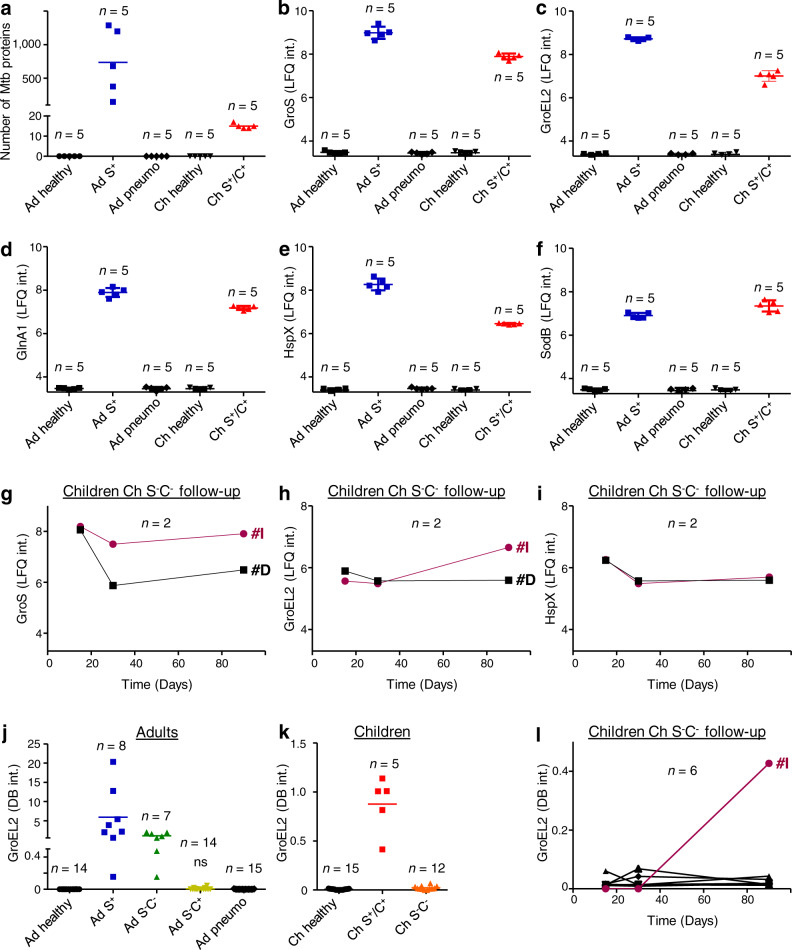
Abundance of *M. tuberculosis* proteins in EBC from TB patients and control individuals listed in Table 1. **a** Number of Mtb proteins detected by proteomic analysis in the corresponding groups. **b**–**i** Abundance of selected Mtb proteins by proteomic analysis. **j**–**l** Abundance of GroEL2 protein by an immunoassay. In **b**–**i**, values are given as the Label-Free Quantification (LFQ) intensity (int.). The noise background intensity was ~3.3 log. In **j**–**l**, values are given in arbitrary units corresponding to intensity (int.) on the Dot Blot (DB) and normalized to levels of GroEL2 in the Mtb cell lysate. In **j** and **k**, unless otherwise stated (ns, not significant), the difference between TB patient groups and controls (healthy, pneumo) was statistically significant (Mann–Whitney *U*-test, two-tailed). Error bars represent SEM. Source data are provided as a Source Data file.

We next analyzed the protein content of EBCs by proteomics. A total number of 1432 Mtb proteins was detected in the 5 individual EBCs analyzed from the smear-positive adults (Ad S^+^) (Supplementary Table 8), with a number of proteins ranging from 147 to 1288 in different patients (Fig. 5a). Only 23 Mtb proteins were detected in smear- or culture-positive children (Ch S^+^/C^+^) (Supplementary Table 8), with a number ranging from 14 to 17 according to the patient (Fig. 5a). In contrast, no Mtb proteins were found in the EBCs of the 5 individuals in each control group (Ad healthy, Ch healthy, Ad pneumo) analyzed (Supplementary Table 8; Fig. 5a; Supplementary Fig. 8). All Mtb proteins detected are listed in Supplementary Data 1. Interestingly, many of them, particularly the most abundant, were previously described to be released in bacterial- and/or host-derived extracellular vesicles^38–41^ (Supplementary Data 2). Protein abundance was determined by Label-Free Quantification (LFQ) (Supplementary Data 2). Changes in abundance (ratio = 1.5) of 656 proteins in adult and 22 proteins in pediatric patients differentiated infected individuals from controls (*P* < 0.05) (Supplementary Data 2; Supplementary Fig. 8). Figure 5 shows the results for selected proteins, GroS (Fig. 5b, g), GroEL2 (Fig. 5c, h), GlnA1 (Fig. 5d), HspX (Fig. 5e, i) and SodB (Fig. 5f). Their abundance was higher in adults, except for SodB, which was slightly higher in children. Among the pediatric patients who were followed during the course of antibiotic treatment (Ch S^-^C^-^, Table 1), the EBCs of 2 (# I and D) were analyzed by proteomics. As observed for LAM and lipids (Figs. 1 and 4), the abundance of all Mtb proteins, other than HspX, remained high, or even increased, during treatment in EBCs of patient #I (Fig. 5g–i and Supplementary Data 2). Finally, we determined the relative abundance of selected Mtb proteins by immunoassay. We tested several monoclonal antibodies against Mtb proteins provided by BEI (www.beiresources.org), including antibodies against GroEL2 (CS-44), HspX (NR-13607), KatG (NR-13793), LpqH (NR-13792) and HBHA (NR-13804). Among these, only the anti-GroEL2 antibody showed enough sensitivity in our dot-blot assay. Abundance of GroEL2 in EBCs (Fig. 5j–l) gave a distribution pattern very similar to that obtained for LAM (Fig. 1) and lipids (Fig. 4), and showed a very good correlation with quantity of LAM (*r* = 0.90, *P* < 0.0001; Supplementary Fig. 6). Although this approach lacked the sensitivity to detect smear-negative patients (Ad S^-^C^+^ and Ch S^-^C^-^), that were under antibiotic treatment for 2 weeks or less (giving positive signals for LAM, MA and TbAd), it allowed us to distinguish the other baseline TB patients from control individuals.

## Discussion

Improving early diagnosis is key to controlling TB burden by decreasing the risk of patient mortality and limiting transmission^1^. Sputum is currently the main sample used for microbiological diagnosis of TB. However sputum-based assays, including microscopic examination, nucleic acid amplification or bacterial culture, have clear limitations, particularly in children and paucibacillary patients^1^. Here, we tested whether EBCs are an accessible sample for the detection of TB with the potential for diagnostic development^3^. Whereas previous studies failed to detect Mtb nucleic acids in EBC of smear- and culture-positive pulmonary TB patients^42,43^, our findings show that assay of bacterial LAM, lipids or proteins efficiently distinguished baseline TB patients. Patients that were smear-negative and culture-negative adults were identified as having active TB using EBC samples. In addition, culture- or smear-positive, and smear-negative and culture-negative, pediatric patients were detected as TB-positive by EBC analysis. The test specifically detected TB patients relative to healthy controls and individuals with community-acquired bacterial pneumonia. Detection of bacterial biomarkers in EBCs fulfills many criteria that are essential for implementation of point-of-care tests: (i) EBC collection is easy, rapid, affordable, non-invasive and does not require specialized personnel; (ii) although exhaled breath may contain viable Mtb^44–46^, EBC samples can be collected in a sterile manner by adding a filter to the collecting device; thus, handling and analysis do not need to be performed in a high-level biosafety laboratory; (iii) Mtb-derived molecules in EBCs are highly abundant and very diverse; they could be assayed with previously developed tools, such as LAM tests developed for urine^5,7,47,48^. This is exemplified by the LAM assay: LAM is ~1000-fold more abundant in EBC than in urine. EBCs are a promising sample for clinical diagnosis and monitoring. However, widespread use still requires standardization in order to reduce the inter- and intra-subject variability observed across studies for non-volatile compound concentration. Thus, the normal physiological ranges of the various potential human biomarkers need to be defined^8,9^. Although important, normalization appears to be less critical for bacterial biomarkers that are likely to serve as positive/negative indicators of infection^9^. Mtb molecules can be readily and specifically detected in EBC samples, potentially allowing an immediate TB diagnosis and an initiation of treatment during the same clinical encounter. In addition, this approach may address key priorities for development of TB diagnostics^3,47,49^ by allowing, (i) detection of TB in children and in paucibacillary specimens, (ii) diagnosis of a higher proportion of pediatric TB cases using a sampling method that children and parents are comfortable with, (iii) screening for community-based case-finding or for triaging patients in the clinic.

To date, the only prognostic marker for end-of-treatment outcome remains culture conversion status, though the sensitivity of this assay is poor^50^. Our data indicate that detection of bacterial biomarkers in EBCs may also provide a tool for real-time monitoring of treatment efficacy, enabling the early detection of disease relapse. The EBC-based analysis would allow clinicians to respond quickly to the therapeutic needs of patients, and may facilitate analysis of efficacy in clinical trials for personalized, anti-TB therapies.

A diverse range of Mtb molecules with unexpected high levels were found in EBCs from TB patients. Whereas D-arabinose derived from LAM was previously found in the range of ~10-40 ng/mL in the urine of TB patients^18^, we detected it in the range of ~15-120 µg/mL in the EBCs of TB patients at baseline. These data suggest that patients exhale a large quantity, between 0.3 and 2.9 mg, of LAM per day. This represents the amount of LAM found in ~10^11^ to 10^12^ CFUs of Mtb grown in broth, and reflects highly metabolically active bacilli. In the present study, we did not investigate EBCs from patients with extrapulmonary TB. Although the amount of LAM or other Mtb molecules might be drastically reduced, the sensitivity of the current tools for LAM detection in the ng/mL range might be sufficient for the diagnosis of extrapulmonary TB through analysis of EBC samples, in those cases where lungs are also affected. Nevertheless, where required, our data provide a basis for the rational improvement of LAM detection tools^5,7^. By performing a full NMR characterization of LAM present in the lungs of TB patients, our data give insight into the molecular moieties that are best targeted for efficient antibody recognition. LAM in EBCs of both adult and children exhibited an overall structure similar to that found in LAM purified from *M. tuberculosis* grown in broth. However, the structure contains significant modifications that are important for the development of diagnostic antibodies. Most particularly, LAM in EBC contains a greatly reduced amount of branched Ara_6_ motifs that are very immunogenic, and a major epitope for anti-LAM antibodies^17,27,51^. This may explain, at least in part, the discrepancies we observed in the LAM quantity values obtained by antibody vs. chemical assays. Generating antibodies that specifically recognize the “non-mature” Ara_5_ motif (Fig. 2), found in both EBC and urine^23^, should significantly improve the sensitivity of LAM detection in these samples. LAM succinylation was not detected in EBCs, although LAM was found to be hyper-succinylated in the lungs of Mtb-infected C3HeB/FeJ mice^23^. LAM in EBC also seems to be devoid of the MTX motif. However, this motif was detected on LAM-derived oligosaccharides from a TB patient’s urine^23^. Accordingly, an immunoassay targeting the MTX motif and tested on urine samples performed well for TB diagnosis^52^. It is worth noting that LAM structure is not specific to Mtb but is shared by other mycobacteria^6^. Assessing LAM levels might be insufficient to ascertain Mtb vs. non-tuberculous mycobacterial infection in some cases, most particularly if only minute amounts are detected. This might be improved by combining detection of LAM with Mtb-specific lipids, such as SGLs or TbAd, or proteins once appropriate antibodies are developed.

From a fundamental biology perspective, EBCs provide an unprecedented window into Mtb lifestyle in human lungs. It represents an invaluable opportunity to understand how the bacterium realigns its metabolism in response to the environments it encounters during infection, a key step for the development of new chemo- and immuno-therapeutic strategies^19^. In addition to structural modifications in LAM mentioned above, we observed a remodeled structure of PIMs, Ac_4_SGLs and PDIM. Similar profiles for Ac_4_SGLs and PDIM with an increased proportion of high mass molecular species have been previously reported for Mtb grown in vitro on propionate or cholesterol as the limiting carbon source, or in lungs of infected mice^33,34^. Although the structure of LAM and lipids in EBC might not be fully representative of all the LAM and lipid molecules produced and circulating in the body of TB patients, our data indicate that Mtb lipid anabolism is increased in the lung environment, which may support bacterial virulence in this tissue^33^. Interestingly, EBCs contain high amounts of free mycolic acids that typify Mtb biofilm growth^36^. Although it has long been a matter of debate, a recent study shows that Mtb biofilms are relevant to human TB^53^ and represent a drug target^36^.

In conclusion, our findings indicate that detection of bacterial molecules in EBCs provides an affordable and rapid approach that seems superior to the sputum assay for early diagnosis of TB disease. Moreover, EBC sampling has potential for community-based case screening and for monitoring treatment efficacy in real-time. In addition, our analysis of TB patient EBCs indicates that these samples can be used to monitor changes in Mtb metabolism during infection. Future work will develop the diagnostic method and potential biomarkers that have been newly identified here in larger scale clinical studies. This will include validating the proof-of-concept data obtained in the present pilot study using larger cohorts and different groups of patients, including HIV-infected individuals, from different regions of the world. As a first step toward a transfer to the field, we are now evaluating the efficacy of diagnosing TB using EBC samples and currently commercialized LAM detection tests^5,7^. However, we conclude that sampling exhaled air is non-invasive and appears to be a powerful approach that overcomes the limitations in existing assays^45,46,54–58^. An EBC-based approach therefore has potentially broad application for following other pulmonary infectious agents.

## Methods

### Patients and control individuals

Adults and pediatric patients with pulmonary TB were recruited through the TB Control Program of the Secretaría de Salud de Medellín and the Secretaría Seccional de Salud y Bienestar Social de Antioquia, Colombia. The diagnostic tests for TB and HIV were performed in the laboratories of health centers where the patients received primary care. Pulmonary TB diagnosis was performed according to the recommendations of Colombia Ministry of Health and Social Protection (https://www.minsalud.gov.co/salud/publica/PET/Paginas/Tuberculosis.aspx), based on international guidelines^13^, and was defined as clinical, and/or radiographic, and/or bacteriological evidence of pulmonary disease resulting in treatment initiation by the diagnosing clinician. After collection of three sputum samples and analysis for acid-fast bacilli smear and mycobacterial culture, adult patients were defined to have culture-positive pulmonary TB if they had Mtb growth in at least one of the three sputum cultures, and to have culture-negative pulmonary TB if they had no Mtb growth in the three initial sputum samples, but clinical and radiographic presentation consistent with TB^13,14^. Smear positivity was defined as having at least one positive acid-fast bacilli smear (Ziehl-Neelsen staining), regardless of quantity, and smear negativity as having no acid-fast bacilli detected in the three initial sputum smears. Pediatric patients were categorized as string-test smear- or culture-positive if they had Mtb growth or direct visualization (Ziehl-Neelsen staining) in string-test samples, and as string test smear- and culture- negative if they had no Mtb growth or direct visualization in string-test samples, but clinical and radiographic presentation consistent with TB, and improvement of symptoms following anti-tuberculous treatment^1,15^.

The first EBC sample was collected at baseline (before or within the first 2 weeks of beginning anti-TB treatment). Additional EBC samples were obtained for children at different time-points during the anti-TB treatment. Subjects participating in the study, or their legal guardians, read and provided written informed consent, as previously approved by the Ethics Committee of the Facultad de Medicina, Universidad de Antioquia. They did not receive any compensation. Exclusion criteria were: patients positive for HIV, diabetes, cancer, autoimmune diseases, immunosuppressive treatment, smoking, or previous TB. These exclusions allowed us to focus on assessing TB in the absence of factors that influence disease or might confound detection. The groups of individuals who participated in this study include:

#### Smear-positive adult patients

Eight patients between 27 and 62 years old, diagnosed with pulmonary TB by direct Ziehl-Neelsen staining and culture of sputum (Table 1). These patients were under anti-TB treatment for 15 days or less.

#### Smear-negative adult patients

Twenty-one patients with at least three sputum samples negative for Ziehl-Neelsen staining (Table 1). Seven patients (aged 19–70) were culture-negative. Fourteen patients (aged 21–68) were either positive for sputum culture or, for four of them, positive for bronchoalveolar lavage (BAL) direct staining or BAL Mtb culture. These fourteen patients were under anti-TB treatment for 15 days or less.

#### Pediatric TB patients

The diagnostic criteria included a history of contact with a tuberculous adult, cough lasting longer than 2 weeks, reactive tuberculin skin test, and radiographic findings compatible with TB^1,15^. The children (aged 6–12) were separated into two groups based on the detection or not of mycobacteria as indicated above and in Table 1. The latter were under an anti-TB treatment regimen for 15 days or less. EBC samples were collected for 6 of these patients 1 and 3 months after treatment.

#### Control patients with pneumonia

Fifteen adults (aged 18–61) who presented with community-acquired pneumonia were included (Table 1). Inclusion criteria were age ≥18, fever (>38 °C), cough, increased respiratory rate or respiratory distress, infiltrates on chest radiographs and bacterial pneumonia confirmed in sputum. Exclusion criteria were immunodeficiency, chronic lung or heart diseases, neoplasia, hospital-acquired pneumonia or viral pneumonia. Twelve patients had pneumonia with consolidation and three without. All of them presented complicated pneumonia, including 8 bacteremias, 4 empyemas, and 3 pleural effusions.

#### Healthy control individuals

Fifteen healthy adult individuals (aged 26–49; Table 1) who did not have respiratory symptoms nor a history of tuberculosis were recruited by the medical and laboratory personnel from the Facultad de Medicina, Universidad de Antioquia. Fifteen healthy children (aged 7–12; Table 1) who had no previous contact with TB patients nor respiratory symptoms were also recruited. The parents consented to participation of children in this study and informed the researchers that the children were BCG vaccinated.

### Exhaled breath condensate (EBC) collection

The EBCs were collected using R-tubes (Respiratory Research, Inc). The R-tube^TM^ is a non-invasive device that allows the collection and condensation of components of the expiration. R-tubes were equipped with a 0.3-µm filter (Respiratory Research, Inc) between the mouthpiece assembly and the condensation cartridge trap to prevent bacilli contaminating EBCs. Subjects breathed through a mouthpiece and a two-way non-rebreathing valve, which also served as a saliva trap. They were asked to breathe at a normal frequency for 15 min. At least 1 mL of sample was collected and immediately frozen at −20 °C. To determine possible saliva contamination in the EBC samples, α-amylase was measured by the Fishman-Doubilet test using a commercial kit and following the manufacturer’s instructions (Abnova, Taipei, Taiwan). EBC samples that were positive for this test were discarded. Accordingly, no α-amylase was detected by proteomic analysis in the EBCs used in the present study, confirming the absence of saliva contamination^59^.

For normalization between individuals, EBCs were lyophilized and adjusted to a volume of 250 µL of LPS-free water.

EBC samples were unique, but measurements were repeated between one and four times.

### Dot-blot immunoassays

EBCs (1 µL) were spotted using the Bio-Dot SF blotting apparatus (Bio-Rad) on a 0.45 µm nitrocellulose membrane (Bio-Rad) previously immersed for 5 min in Tris-buffered saline (TBS). LAM purified from *M. tuberculosis* H37Rv^21,60^ or a Mtb cell lysate (obtained by sonication with probe) were used as standards and deposited at different amounts (in 1 µL). The membrane was first blocked for 2 h at RT in TBS with 0.01% Tween-20 and 3% skimmed milk powder. It was subsequently incubated overnight at 4 °C with the primary antibodies, mouse IgG3 anti-LAM antibody (CS-35, BEI Resources; dilution of 1:200) or mouse IgG2b anti-GroEL2 (CS-44, BEI Resources; dilution of 1:1000), in TBS supplemented with 1.5% skimmed milk powder. Horseradish peroxidase (HRP)-conjugated secondary antibodies, goat anti-mouse IgG3-HRP (ThermoFisher; dilution of 1:2000) or goat anti-mouse IgG2b-HRP (Invitrogen; dilution of 1:5000), in TBS supplemented with 1.5% skimmed milk powder were incubated with the membrane for 2 h at RT. After each antibody incubation step, the membrane was washed 3 times with TBS supplemented with 0.01% Tween-20. Membranes were processed to reveal signal using Amersham ECL Prime™ and images were captured using the ChemiDoc System (Bio-Rad). Quantification of dot density was performed using Image Lab^TM^ Software (Bio-Rad; version 6.0.1 build 34).

The following monoclonal antibodies, provided by BEI Resources, were also tested: anti- HspX (IT-20; dilution of 1:100), anti-KatG (IT-57; dilution of 1:200), anti-LpqH (IT-54; dilution of 1:200), anti-HBHA (α-HBHA; dilution of 1:50).

### Monosaccharide analysis and quantification by capillary electrophoresis monitored by laser-induced fluorescence (CE-LIF)

EBCs (12 to 25 µL) were submitted to total acid hydrolysis using 2 M trifluroacetic acid for 2 h at 110 °C, in the presence of 1 nmol mannoheptose (Sigma) as an internal standard. The samples were dried and mixed with 0.3 µL of 0.2 M 8-aminopyrene-1,3,6-trisulfonic acid (APTS; Sigma) in 15% acetic acid and 0.3 µL of a 1 M sodium cyanoborohydride solution dissolved in tetrahydrofuran. The reaction mixture was heated at 55 °C for 90 min and subsequently quenched by the addition of 20 µL of water. Monosaccharide APTS-derivatives were analyzed and quantified by capillary electrophoresis monitored by laser-induced fluorescence on a P/ACE capillary electrophoresis system (Beckman Instruments, Inc.). Separations were performed using an uncoated fused-silica capillary column (Sigma) of 50-µm internal diameter and 40-cm effective length (47-cm total length). Analyses were carried out at a temperature of 25 °C, in the reverse mode, with an applied voltage of 20 kV using 1% acetic acid (w/v)−30 mM triethylamine, pH 3.5, as a running electrolyte^25^.

### Preparation of LAM-enriched fractions from EBC pools

The LAM-enriched fraction from EBC pools was prepared using an approach similar to that described for LAM purification from bacteria^61^. Lipids were removed by chloroform/methanol extraction (partition chloroform/methanol/water 1.25:1.25:1). The aqueous phase was dried and submitted to digestion by DNAse (D4263, Sigma), RNAse (R4875, Sigma) and a cocktail of proteases (α-chymotrypsin (C4129, Sigma-Aldrich), *Streptomyces griseus* proteases (P8811, Sigma) and trypsin (T3914, Sigma-Aldrich)) to remove nucleic acids and proteins. The digested solution was dialyzed against water (MWCO 6–8 kDa) and dried, resulting in a fraction enriched in LAM.

### Monosaccharide analysis by gas chromatography coupled to mass spectrometry (GC-MS)

Monosaccharides were analyzed as their trimethylsilyl (TMS) derivatives after total acid hydrolysis (2 M trifluroacetic acid for 2 h at 110 °C) of EBC pools (5 mg), drying, and derivatization with pyridine/hexamethyldisilazane/chlorotrimethylsilylane, 4:2:1, v/v/v, for 15 min at room temperature, and injection in GC-MS in electron ionization mode.

To determine the absolute configuration of arabinose, EBC pools (5 mg) were octanolysed using 2 M trifluoroacetic in (R)-(−)−2-butanol (Sigma) for 16 h at 110 °C^62^. Octyl-glycosides were dried and converted into their TMS derivatives, as detailed above, before analysis by GC-MS and comparison with D-Ara and L-Ara standards submitted to the same procedure.

GC-MS was performed on a Trace 1300 Thermo GC equipped with a TG-SQC column (15-m, 0.25 mm inner diameter, 0.25 µm film thickness) coupled to an ISQ quadrupole mass spectrometer. The carrier gas was helium at a flow rate of 1 mL/min. The injector temperature was 250 °C and the temperature separation program was from 100 to 300 °C, at a speed of 15 °C/min.

### Mannose cap and western blot analyses of LAM-enriched fractions

For mannose cap analysis and quantification, LAM-enriched fractions (100 µg) were subjected to mild acid hydrolysis using 0.1 M HCl for 20 min at 110 °C, in the presence of 0.1 nmol mannoheptose (Sigma) as an internal standard. Samples were dried, derivatized by APTS and analyzed by capillary electrophoresis as described above^25^.

For western blot analysis, LAM-enriched fractions (2–10 µg) were subjected to SDS–PAGE (15% separating gel)^61^ and transferred to a nitrocellulose membrane, which was subsequently probed with CS-35 anti-LAM antibody as described above.

### MALDI-TOF analysis

MALDI-TOF analyses were performed on an AB Sciex TOF/TOF 5800 mass spectrometer using the reflectron mode^29,63^. Ionization was achieved by irradiation with an Nd:YAG laser (349 nm) operating with a pulse rate of 400 Hz. The laser intensity was set at 3500 and continuous stage motion was used with a velocity of 600 µm/s. Data was acquired in the negative ion mode. Typically, spectra from 2500 to 5000 laser shots were summed to obtain the final spectrum. 2,5-dihydroxybenzoic acid (Sigma) was used as the matrix at a concentration of 10 mg/mL in chloroform/methanol 9:1 or 1:1.

### NMR analysis

NMR spectra were recorded at 305 K without sample spinning on a Bruker 600 MHz avance III HD (1H) equipped with a TCi cryoprobe (Bruker Biospin, Germany). LAM-enriched fractions were exchanged in D_2_O (D, 99.97 % from Euriso-top, Saint-Aubin, France), with intermediate lyophilisation, and then dissolved in 0.5 mL D_2_O. Samples were analyzed in 200 × 5 mm UL-5 NMR tubes (Euriso-Top, Saint-Aubin, France). Proton and carbon chemical shifts are expressed in parts per million and referenced relative to internal acetone signals at δ_H_ 2.225 and δ_C_ 31.45. ^1^H-^13^C correlation spectra were acquired in the echo/anti-echo acquisition mode recorded in the proton-detected mode using the “hsqcetgpsisp2.2” sequence from the Topspin 3.5 PL7 software (Bruker Biospin, Germany). The 2D ^1^H-^13^C spectra were recorded with a spectral width of 22,636 Hz in ^13^C and 7211 Hz in ^1^H dimensions in order to collect a 2048 × 512 point data matrix with 16 scans per t1 value expanded to 4096 × 1024 by zero filling. The relaxation delay was 1.5 s. A sine bell window shifted by *π*/2 was applied in both dimensions^20,21^.

### Supercritical fluid chromatography (SFC)-MS lipidomic analysis

EBCs (30 µL) were mixed with 4 ng of 1,2-ditridecanoyl-*sn*-glycero-3-phosphocholine (Avanti Polar Lipids), used as an internal standard allowing the quality control of extraction and normalization for comparative analyses, 12 µL of water and 105 µL of chloroform/methanol 1:1 in a glass vial. After vortexing and phase separation, the organic phase was recovered for SFC-MS analysis at the MetaToul Lipidomic Core Facility (I2MC, Inserm 1048, Toulouse, France) instrument. The Ultra-Performance Convergence Chromatography (UPC2) was coupled on-line to a Xevo G2-XS time of flight (QTOF) (Waters, Milford, MA, USA), equipped with ESI under the control of MassLynx software, version 4.1. The analysis was performed in both ionization modes (positive and negative) in two separate runs. One microliter of lipid extract was then injected into the ACQUITY UPC2 Torus Diol column (100 × 3.0 mm inner diameter (i.d.), particle size: sub-1.7 µm, Waters) at 48 °C. The lipid families were separated using a gradient of supercritical CO_2_/CH_3_OH (1.25 mg/mL ammonium formate) (1%–50%) at a 1.4 mL/min flow rate and using an active back pressure regulator (ABPR) of 1.900 pounds per square inch (psi). The make-up solvent was CH_3_OH used at 0.25 mL/min. The source parameters were set as follows: source temperature 120 °C; the capillary voltage 3.2 kV in positive mode and −3 kV in negative mode; the desolvation gas flow rate 690 mL/h in positive mode and 740 mL/h in negative mode; the cone gas flow 40 L/h; the desolvation temperature 420 °C in positive mode and 390 °C in negative mode. The analyses were performed in MS full scan in centroid mode from 50 to 3000 Da. Mtb lipids families were detected with characteristic retention times and previously reported^64^ acyl-form profiles and accurate *m/z*.

For each lipid family, a chromatogram of the main representative acyl-form was extracted at the corresponding *m/z* within 20 ppm. Area of the extracted ion chromatograms (EIC) was measured using Waters MassLynx™ Software v. 4.2. Peak area integration was done with the following parameters: window size (scans) ±3, number of smooths 2 and a mean smoothing method. The relative abundance of each lipid family was determined relatively to 1,2-ditridecanoyl-*sn*-glycero-3-phosphocholine used as an internal standard, and calculated as the ratio of measured EIC areas of lipids vs. internal standard.

### Sample preparation for label-free proteomics analysis

For each sample, 35 µg of dried protein extracts were solubilized with 25 µL of 5% SDS. Proteins were submitted to reduction and alkylation of cysteine residues by addition of TCEP and chloroacetamide to a final concentration respectively of 10 mM and 40 mM. Protein samples were then processed for trypsin digestion on S-trap Micro devices (Protifi) according to the manufacturer’s protocol, with the following modifications: precipitation was performed using 216 µL S-Trap buffer, 1.5 µg trypsin was added per sample for digestion in 25 µL TEAB 50 mM pH 8.

### NanoLC-MS/MS analysis of proteins

Tryptic peptides were resuspended in 35 µL of 2% acetonitrile and 0.05% trifluoroacetic acid and analyzed by nano-liquid chromatography (LC) coupled to tandem MS, using an UltiMate 3000 system (NCS-3500RS Nano/Cap System; Thermo Fisher Scientific) coupled to an Orbitrap Q-HFX mass spectrometer (Thermo Fisher Scientific). Around 5 µg of each sample was loaded on a C18 precolumn (300 µm inner diameter × 5 mm, Thermo Fisher Scientific) in a solvent made of 2% acetonitrile and 0.05% trifluoroacetic acid, at a flow rate of 20 µL/min. After 5 min of desalting, the precolumn was switched online with the analytical C18 column (75 µm inner diameter × 50 cm, Acclaim PepMap 2 µm C18, Thermo Fisher Scientific) equilibrated in 95% solvent A (5% acetonitrile, 0.2% formic acid) and 5% solvent B (80% acetonitrile, 0.2% formic acid). Peptides were eluted using a 10%–45% gradient of solvent B over 60 min at a flow rate of 350 nL/min. The mass spectrometer was operated in data-dependent acquisition mode with the Xcalibur software (version 4.3.73.11). MS survey scans were acquired with a resolution of 60,000 and an AGC target of 3 × 10^6^. The 12 most intense ions were selected for fragmentation by high-energy collision-induced dissociation, and the resulting fragments were analyzed at a resolution of 30,000, using an AGC target of 1 × 10^5^ and a maximum fill time of 54 ms. Dynamic exclusion was used within 30 s to prevent repetitive selection of the same peptide.

### Bioinformatics analysis of proteomic datasets

Raw MS files were processed with the Mascot software (version 2.7.0) for database search and Proline^65^ for label-free quantitative analysis (version 2.1.2). Data were searched against MTB_H37Rv_native and Human entries of the UniProtKB protein database (release Swiss-Prot+TrEMBL 2020_10, 4080 entries and release Swiss-Prot 2020_10, 20,385 entries, respectively). Carbamidomethylation of cysteines was set as a fixed modification, while oxidation of methionine was set as variable modifications. Specificity of trypsin/P digestion was set for cleavage after K or R, and two missed trypsin cleavage sites were allowed. The mass tolerance was set to 10 ppm for the precursor and to 20 mmu in tandem MS mode. Minimum peptide length was set to 7 amino acids, and identification results were further validated in Proline by the target decoy approach using a reverse database at both a PSM and protein false-discovery rate of 1%. For label-free relative quantification of proteins across biological replicates and conditions, cross-assignment of peptide ions peaks was enabled inside a group with a match time window of 1 min, after alignment of the runs with a tolerance of +/− 600 s.

Median Ratio Fitting computes a matrix of abundance ratios calculated between any two runs from ion abundances for each protein. For each pair-wise ratio, the median of the ion ratios is then calculated and used to represent the protein ratio between these two runs. A least-squares regression is then performed to approximate the relative abundance of the protein in each run in the dataset. This abundance is finally rescaled to the sum of the ion abundances across runs.

A student *t*-test (two-tailed *t*-test, equal variances) was then performed on log2 transformed values and followed by Adjusted Benjamini–Hochberg (ABH) correction to analyze differences in protein abundance in all biologic group comparisons. Significance level was set at *P* = 0.05, and ratios were considered relevant if higher than +/− 2.

The mass spectrometry proteomics data have been deposited to the ProteomeXchange Consortium via the PRIDE partner repository with the dataset identifier PXD028477.

### Statistics and reproducibility

EBC samples are unique. Values shown in graphs or tables are the mean of one to several independent measurements (technical replicates) on each individual EBC sample. Statistical differences between TB patient groups and controls (healthy, pneumo) were assessed using a two-tailed Mann–Whitney *U*-test and a α level of 0.05. Correlations between the different Mtb biomarkers were determined by Pearson correlation test. Analyses, including ROC, were done using Prism 5.0 (GraphPad Software). Leave-One-Out cross-validation analysis was carried out with R software (version 4.2.1)^66^, using the *caret* package (version 6.0-93) for the cross-validation^67^, and *tidyverse* (version 1.3.2) for data wrangling and graphical output^68^.

### Reporting summary

Further information on research design is available in the Nature Portfolio Reporting Summary linked to this article.

## Supplementary information

Supplementary information

Description to Additional Supplementary Information

Supplementary Data 1

Supplementary Data 2

Reporting Summary

## Data Availability

Raw data are provided as a Source Data file. The mass spectrometry proteomics data were searched against MTB_H37Rv_native and Human entries of the UniProtKB protein database (release Swiss-Prot+TrEMBL 2020_10, 4080 entries and release Swiss-Prot 2020_10, 20,385 entries, respectively), and have been deposited to the ProteomeXchange Consortium via the PRIDE partner repository with the dataset identifier PXD028477. Source data are provided with this paper.

## Source data

Source data File
