# Supplementary material for: A Mycobacterium tuberculosis fingerprint in human breath allows tuberculosis detection
Source: Nat Commun. 2022 Dec 14;13:7751. doi: 10.1038/s41467-022-35453-5 (PMC9751131; doi:10.1038/s41467-022-35453-5)

Supplementary information for

A *Mycobacterium tuberculosis* fingerprint in human breath allows tuberculosis detection

Sergio Fabián Mosquera-Restrepo, Sophie Zuberogoïtia, Lucie Gouxette, Emilie Layre, Martine Gilleron, Alexandre Stella, David Rengel, Odile Burlet-Schiltz, Ana Cecilia Caro, Luis F. Garcia, César Segura, Carlos Alberto Peláez Jaramillo, Mauricio Rojas and Jérôme Nigou

Supplementary Figures 1 to 8
Supplementary Tables 1 to 8
Supplementary references
Legend of Supplementary Data 1 & 2
Uncropped scans of Supplementary Fig. 2
Uncropped scan of Supplementary Fig. 7

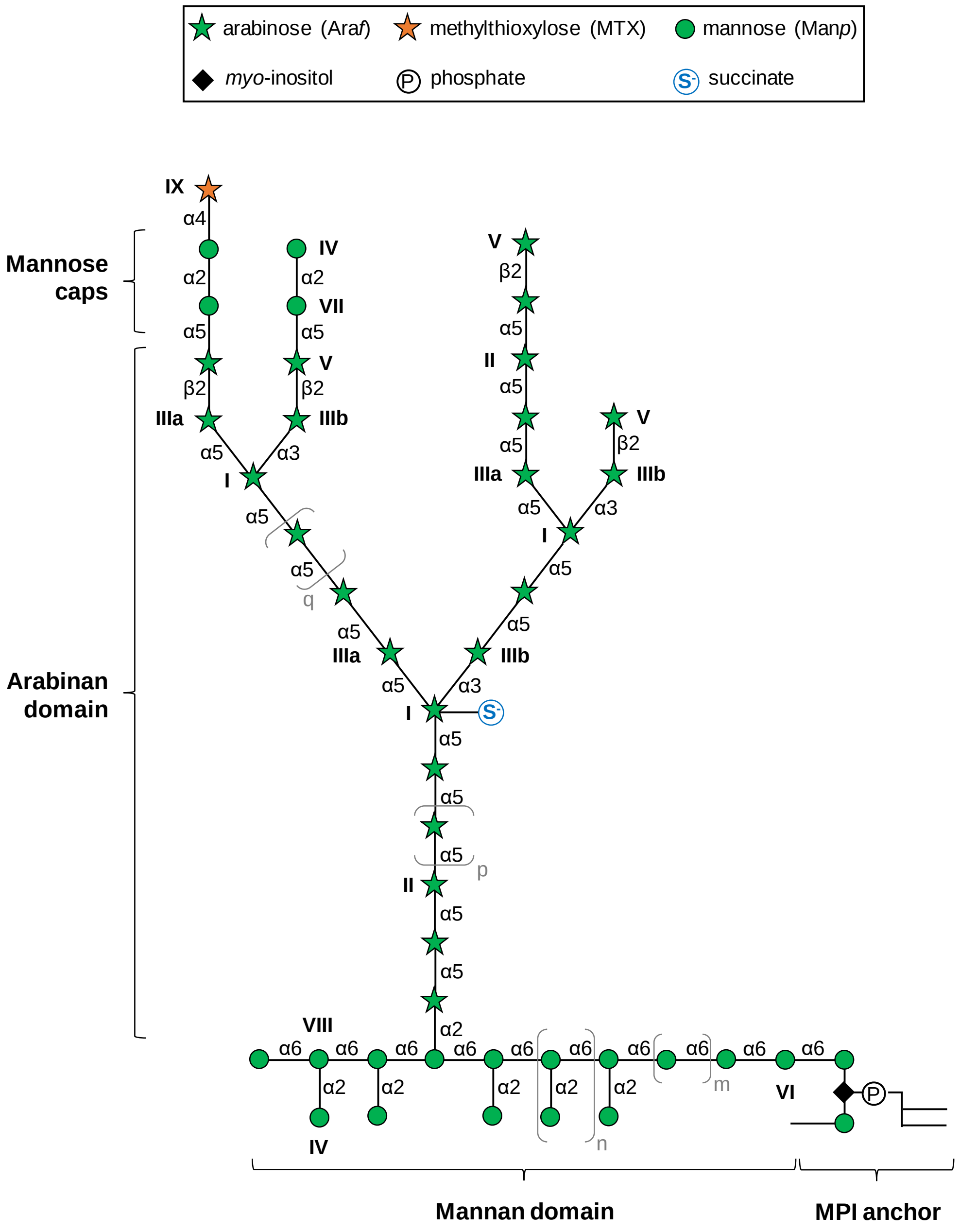

Supplementary Fig. 1. Schematic structure of LAM.
Ara*f*, arabinofuranose; Man*p*, mannopyranose; MPI, mannosyl-phosphatidyl-*myo*-inositol.

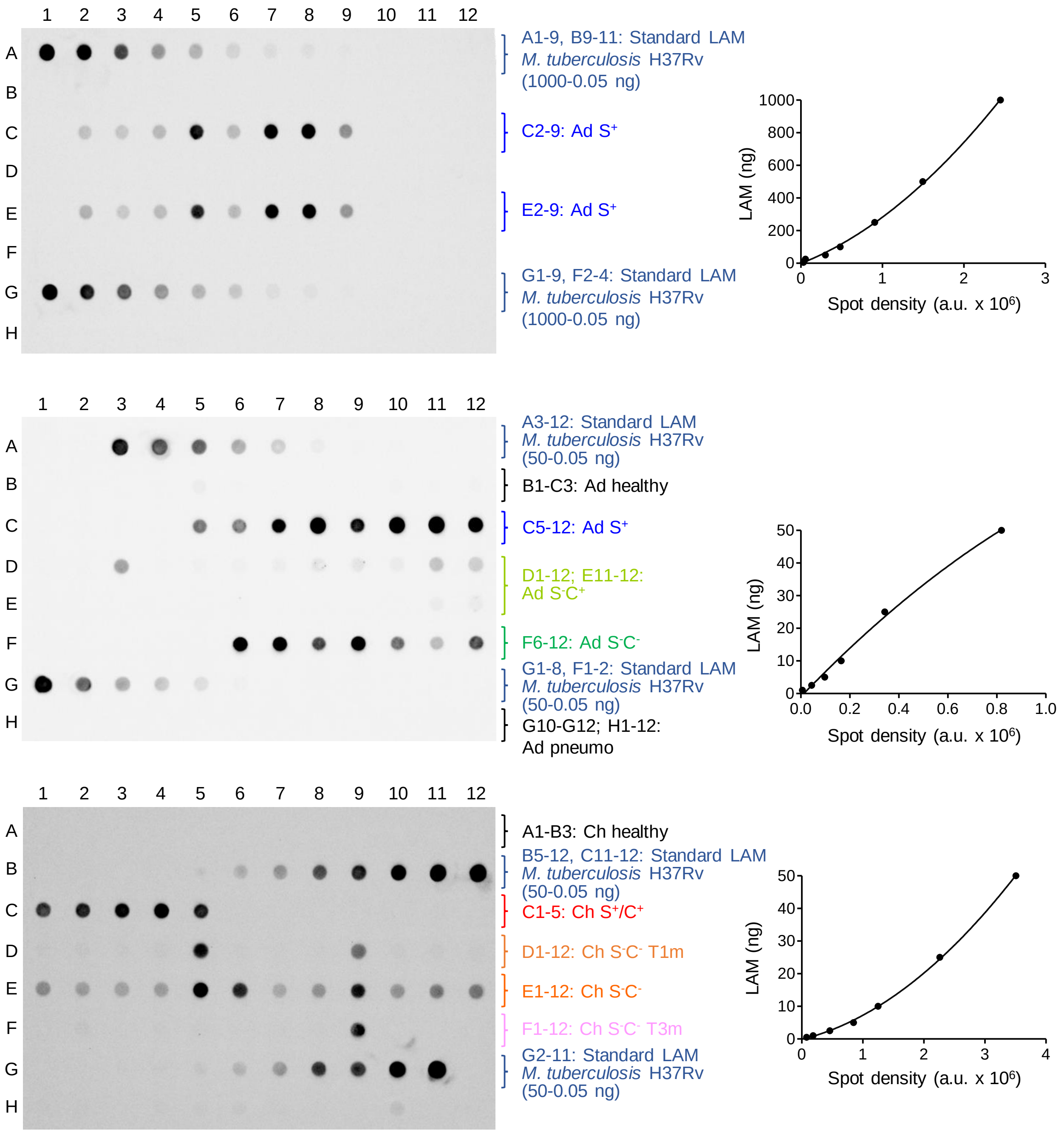

Supplementary Fig. 2. Immunoassay for LAM quantification using CS-35 anti-LAM antibody.
Examples of dot-blot images and calibration curves are shown. Spot density was dermined using Image Lab^{TM} Software (Bio-Rad). T1m, T3m, after 1 and 3 months of antibiotic treatment respectively.

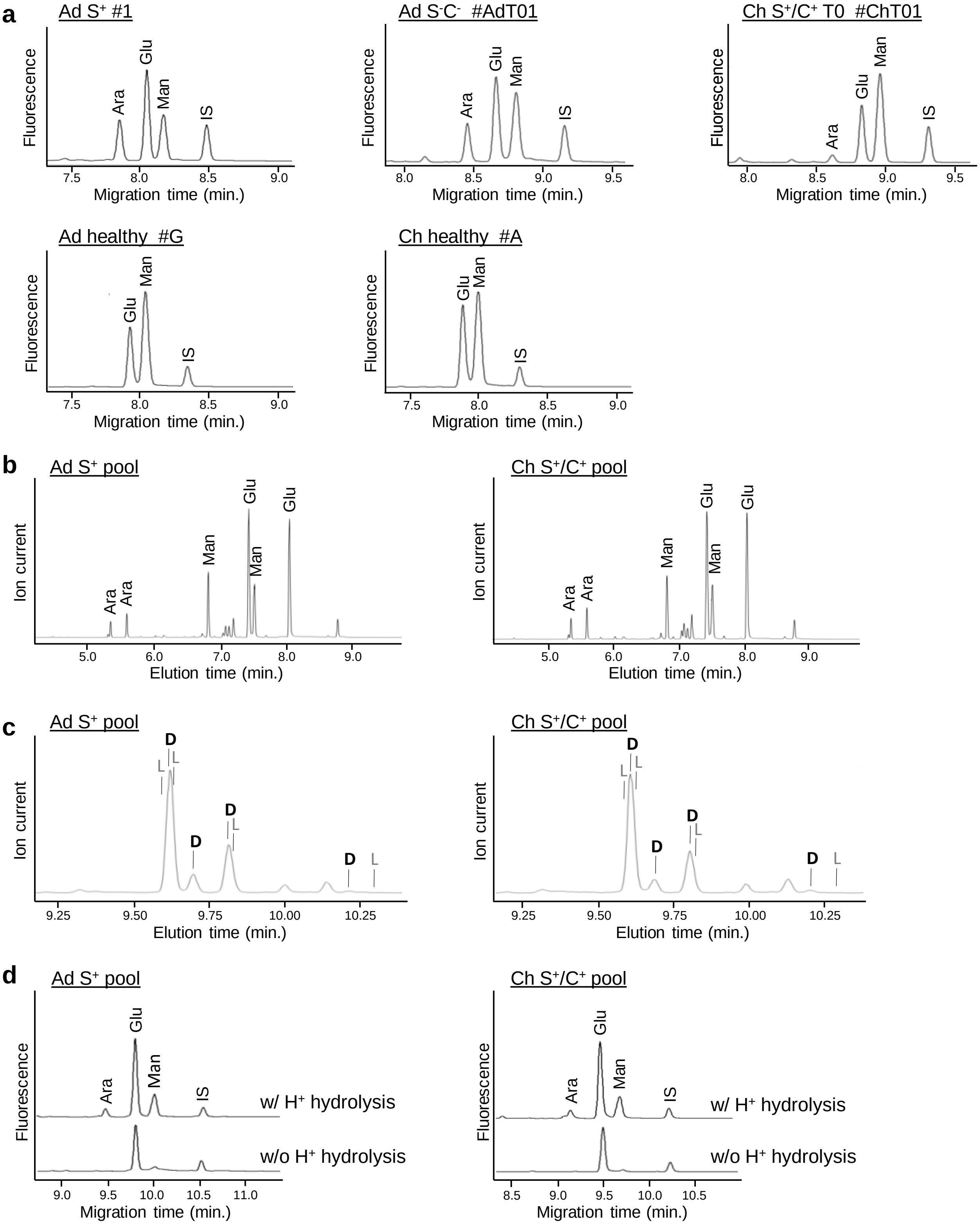
a
Ad S^+ #1
Ad S^-C^- #AdT01
Ch S^+/C^+ T0 #ChT01
Ad healthy #G
Ch healthy #A
Fluorescence
Migration time (min.)
Ara
Glu
Man
IS
b
Ad S^+ pool
Ch S^+/C^+ pool
Ion current
Elution time (min.)
c
Ad S^+ pool
Ch S^+/C^+ pool
D
L
Ion current
Elution time (min.)
d
Ad S^+ pool
Ch S^+/C^+ pool
w/ H^+ hydrolysis
w/o H^+ hydrolysis
Fluorescence
Migration time (min.)

Supplementary Fig. 3. Chemical detection of arabinose and other monosaccharides in EBCs.

a) Analysis of monosaccharides by CE-LIF upon total acid hydrolysis and fluorescent labeling by 8-Aminopyrene-1,3,6- trisulfonate (APTS). Representative electropherograms obtained for selected patients/individuals in different groups are shown.

b) Analysis of monosaccharides by GC/MS upon total acid hydrolysis and trimethylsilylation.

c) Analysis of arabinose configuration by GC/MS upon butanolysis and trimethylsilylation.

d) Analysis of free monosaccharides without (w/o) acid hydrolysis by CE-LIF upon fluorescent labeling by APTS.

In b, c and d, analyses were performed on pooled EBCs collected from 50 adults (Ad S^+ pool) or 50 children (Ch S^+/C^+ pool) TB patients (Extended Data Table 1).

Ara, Glu, Man, and IS correspond to arabinose, glucose, mannose and internal standard (mannoheptose) derivatives respectively; D, L indicate the elution time of the trimethylsilylated R-(-)-2-butyl glycosides of D- and L-arabinose respectively.

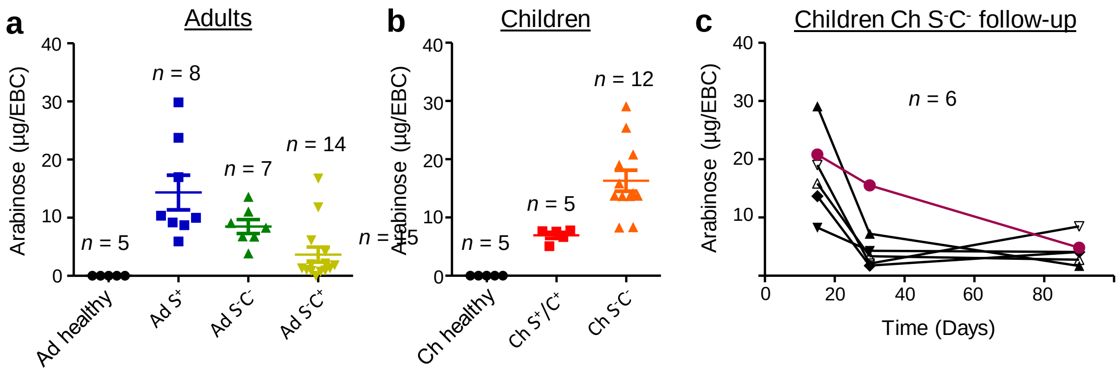

Supplementary Fig. 4. Quantification of arabinose as a proxy of LAM in EBC from TB patients and control individuals listed in Table 1.

Quantity of arabinose per EBC from adults (a) and children (b, c) was determined by acid hydrolysis and CE-LIF analysis. In a and b, the difference between TB patient groups and controls (healthy, pneumo) was statistically significant (Mann-Whitney *U*-test, two-tailed). Error bars represent SEM. Source data are provided as a Source Data file.

a

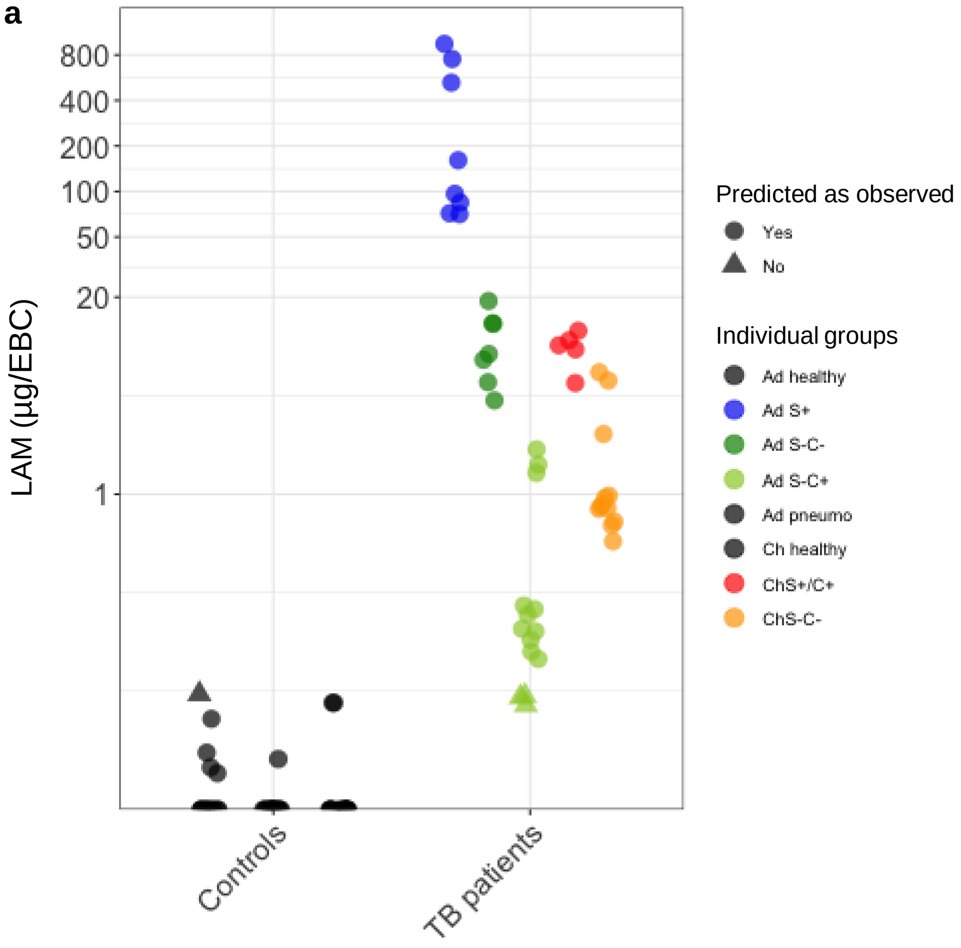

b

		Actual condition	
		Controls	TB patients
**Prediction outcome**	Controls	44	3
	TB patients	1	43

Supplementary Fig. 5. Graphical visualization of the Leave-One-Out cross-validation prediction (a) and tabular comparison between actual individual condition and Leave-One-Out cross-validation-predicted condition (b).

a) Quantity of LAM in EBC samples for control individuals and TB patients are shown. Symbol colors correspond to individual groups. Circles correspond to individuals whose actual condition was correctly predicted by the Leave-One-Out cross-validation (LOOCV), whereas triangles correspond to individuals whose actual condition was not correctly predicted by the LOOCV.

b) Columns refer to actual individual condition and rows to the LOOCV prediction. Numbers correspond to the frequencies of cross-grouping between actual and LOOCV-predicted conditions.

Ad, adult; Ch, child.

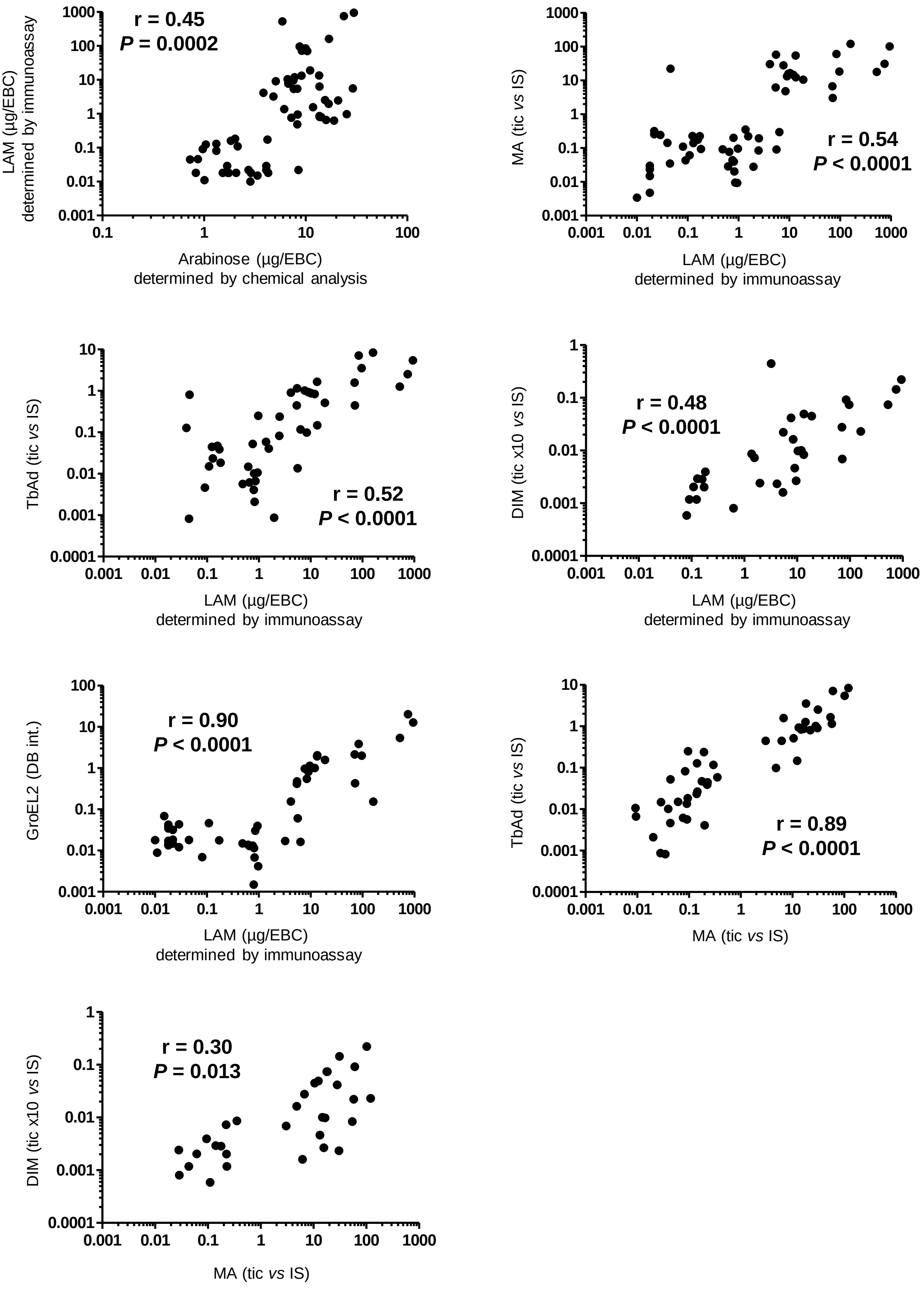

Supplementary Fig. 6. Correlation between the measured abundance of the different Mtb molecules in individual EBCs. Pearson's r and *P* values (two-tailed) are provided. A total of 64 EBCs was collected for TB patients (46 at baseline, 12 and 6 after 1 and 3 months of antibiotic treatment respectively for S^-C^- pediatric patients).

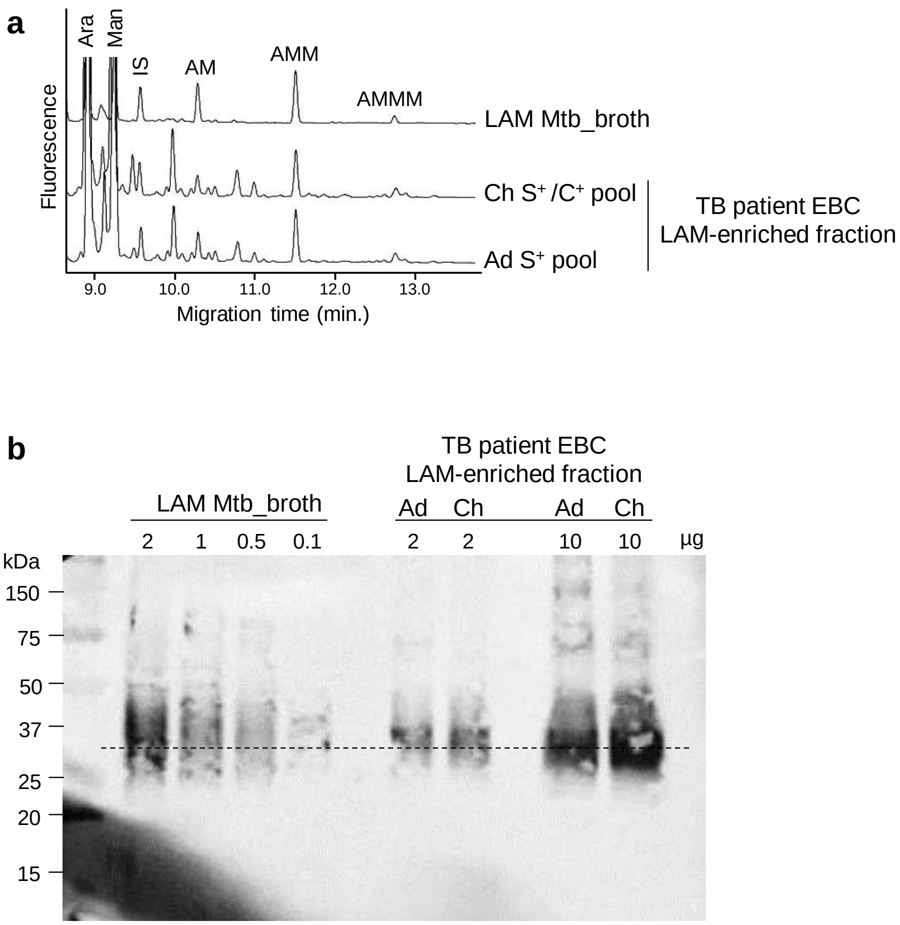

Supplementary Fig. 7. LAM in EBC bear mannose caps (a) and exhibit a lower apparent MW (b).
a) Analysis of mannose caps by CE-LIF upon mild acid hydrolysis and fluorescent labeling by 8-Aminopyrene-1,3,6-trisulfonate. IS, internal standard, mannoheptose-APTS; AM, Man*p*-(α1→2)-Ara-APTS; AMM, Man*p*-(α1→2)-Man*p*-(α1→2)-Ara-APTS; AMMM, Man*p*-(α1→2)-Man*p*-(α1→2)-Man*p*-(α1→2)-Ara-APTS.
b) Western blot probed with CS-35 anti-LAM antibody. The different fractions were loaded according to the quantity of arabinose determined by CE-LIF quantification (Extended Data Table 2), 0.1-2 µg for Mtb_broth LAM and 2-10 µg for LAM-enriched fraction from Ad S^+ pool and Ch S^+/C^+ pool.
Ad, adult; Ch, child.
Data are representative of 2 independent experiments.

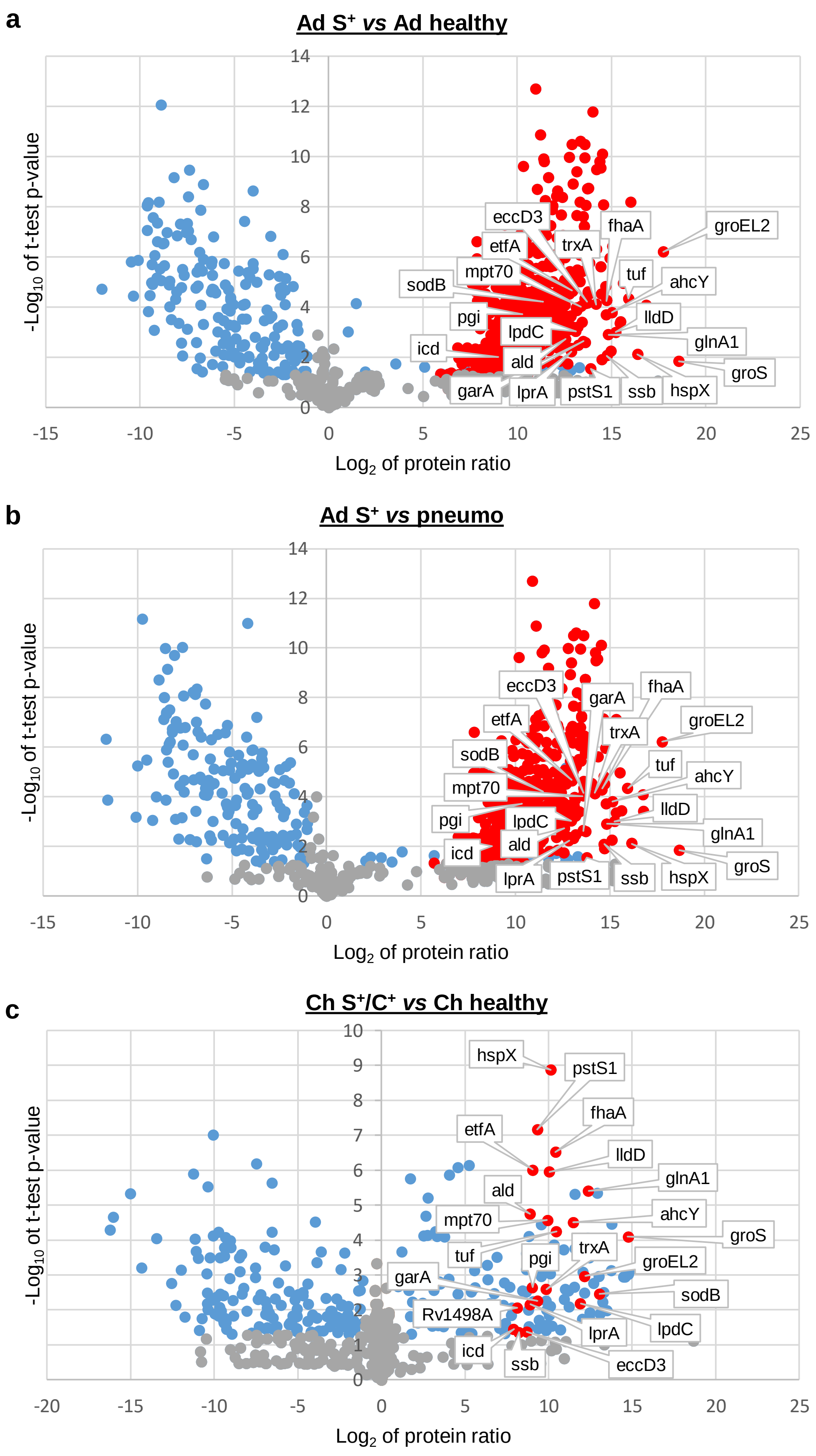

Supplementary Fig. 8. Volcano plot of log significance (paired t-tests) versus log paired ratio of changes in protein abundance.

Grey circles represent identified proteins not showing significant differences whereas red and blue circles represent Mtb and human proteins respectively with significantly different abundances between the two indicated groups ($p < 0.05$; ratio = 1.5). A Student t-test (two-tailed t-test, equal variances) was performed on log2 transformed values and followed by Adjusted Benjamini–Hochberg (ABH) correction to analyze differences in protein abundance in all biologic group comparisons.

Supplementary Table 1. Clinical and demographic characteristics of the adult and pediatric TB patients whose EBC was pooled.

		Adults	Children
		**Smear-positive**	**String test smear- or culture-positive**
**Number of patients**		50	50
**Gender M/F**		24/26	30/20
	**Median**	38	9
**Age**	**Minimal**	15	6
	**Maximal**	60	12
**TST[a] (positive/performed)**		44/50	6/50
**TST Diameter (mm)[b]**		12 ± 8 (0-25)	10 ± 2 (0-16)
**BCG scar (positive/total)**		40/50	6/50
**Primary treatment**		Isoniazid[c] Rifampicin[c]	Isoniazid[c] Rifampicin[c]
**Time of EBC collection**		Before antibiotic treatment	Before antibiotic treatment
**Label**		**Ad S^+ pool**	**Ch S^+/C^+ pool**

[a] tuberculin skin test
[b] mean ± SD (range)
[c] antibiotic treatment started after EBC collection

Supplementary Table 2. Global composition of EBC pools and LAM-enriched fractions

	Mass (mg)	Ad S^+ pool	Ch S^+/C^+ pool
EBC	Total[a]	630	720
	Proteins[b]	172 (27%)	189 (26%)
	Lipids[c]	9.4 (1.5%)	14.3 (2%)
	Arabinose[d]	0.67 (0.1%)	0.76 (0.1%)
	Glucose[d]	6.7[e]	6.6[e]
	Mannose[d]	2.9[f]	3.1[f]
LAM-enriched fraction	Total	11.3	27
	Arabinose[d]	0.18	0.36
	Glucose[d]	0.09	0.24
	Mannose[d]	0.83	1.8

[a], determined by weighing
[b], determined by BCA protein assay
[c], determined after extraction of lipids by organic solvents and weighing
[d], determined after total acid hydrolysis, or not, and quantification by CE-LIF
[e], 60% of Glu was found as a free monosaccharide (detected without total acid hydrolysis)
[f], 10% of Man was found as a free monosaccharide (detected without total acid hydrolysis)

Supplementary Table 3. Anomeric ^{1}H and ^{13}C NMR chemical shifts of LAM in EBC pools measured at 298 K in D_2O.

Differences between LAM in EBC and LAM purified from *M. tuberculosis* H37Rv grown in both (Mtb_broth) are highlighted in blue. Ara*f*, arabinofuranose; Man*p*, mannopyranose; MTX, methylthioxylose.

Residue		Ad S^+ pool	Ch S^+/C^+ pool	LAM Mtb_broth
I, 3,5-α-Ara*f*	^{13}C	110.2	110.2	110.1
	^{1}H	5.13	5.13	5.14
II, 5-α-Ara*f*	^{13}C	109.9	109.9	109.8
	^{1}H	5.18	5.18	5.19
	^{13}C	110.3	110.3	110.2
	^{1}H	5.10	5.10	5.11
IIIa, 2-α-Ara*f*→5	^{13}C	108.4	108.4	108.4
	^{1}H	5.19	5.19	5.21
IIIb, 2-α-Ara*f*→3	^{13}C	108.2	108.2	108.1
	^{1}H	5.26	5.26	5.28
IV, t-α-Man*p*	^{13}C	104.9	104.9	104.9
	^{1}H	5.06	5.06	5.06
V, t-β-Ara*f*	^{13}C	103.4	103.5	103.3/103.2
	^{1}H	5.16	5.16	5.18/5.30
VI, 6-α-Man*p*	^{13}C	102.3	102.2	102.3
	^{1}H	4.92	4.92	4.94
VII, 2-α-Man*p*	^{13}C	101.0	101.0	100.9
	^{1}H	5.15	5.16	5.19
VIII, 2,6-α-Man*p*	^{13}C	100.9	100.9	100.9
	^{1}H	5.13	5.13	5.17
IX, MTX	^{13}C	ND	ND	105.3/105.0
	^{1}H	ND	ND	5.48/5.43
Xa, t-α-Araf→5	^{13}C	110.6	110.6	ND
	^{1}H	5.04	5.04	ND
Xb, t-α-Araf→3	^{13}C	109.7	109.7	ND
	^{1}H	5.24	5.24	ND

ND, not detected

Supplementary Table 4. List of the [M-H]⁻ ions of the PIM and corresponding major molecular species detected by MALDI-TOF mass spectrometry in Ch S^+/C^+ pool.

Peak assignment was performed according to previous studies[1-3]. The last column indicates the number and the fatty acyl chains (C_{16}, palmitoyl; C_{18}, stearoyl; C_{19}, tuberculostearoyl, i.e. 10-methyl-stearoyl) esterifying the PIM molecules (PIM_1, PIM_2, PIM_3, or PIM_6: mono-, di-, tri-, or hexa-mannosides: respectively).

In red are indicated the molecular species found in trace amount only in Mtb_broth and that show increased abundance in EBCs.

Measured *m/z* of $[M-H]^-$	Calculated *m/z* of $[M-H]^-$	Molecular formula of M	Relative Intensity (%)	Species	Fatty acyl chains
851.6	851,5655	$C_{44}H_{85}O_{13}P$	n.d.[a]	PI	$1C_{16}$, $1C_{19}$
1133.6	1133,6242	$C_{53}H_{99}O_{23}P$	3.9	PIM_2	$2C_{16}$
1161.7	1161,6555	$C_{55}H_{103}O_{23}P$	3.4	PIM_2	$1C_{16}$, $1C_{18}$
1175.7	1175,6711	$C_{56}H_{105}O_{23}P$	8.3	PIM_2	$1C_{16}$, $1C_{19}$
1189.7	1189,6868	$C_{57}H_{107}O_{23}P$	1.8	PIM_2	$2C_{18}$
1203.7	1203,7024	$C_{58}H_{109}O_{23}P$	1.8	PIM_2	$1C_{18}$, $1C_{19}$
1399.9	1399,8852	$C_{71}H_{133}O_{24}P$	2.2	Ac_1PIM_2	$2C_{16}$, $1C_{18}$
1413.9	1413,9008	$C_{72}H_{135}O_{24}P$	15.2	Ac_1PIM_2	$2C_{16}$, $1C_{19}$
1427.9	1427,9165	$C_{73}H_{137}O_{24}P$	3.0	Ac_1PIM_2	$1C_{16}$, $2C_{18}$
1441.9	1441,9321	$C_{74}H_{139}O_{24}P$	3.5	Ac_1PIM_2	$1C_{16}$, $1C_{18}$, $1C_{19}$
1456.0	1455,9478	$C_{75}H_{141}O_{24}P$	2.2	Ac_1PIM_2	$1C_{16}$, $2C_{19}$
1476.1	1476,0620	$C_{81}H_{153}O_{20}P$	3.0	Ac_2PIM_1	$3C_{16}$, $1C_{18}$
1504.1	1504,0933	$C_{83}H_{157}O_{20}P$	5.1	Ac_2PIM_1	$2C_{16}$, $2C_{18}$
1546.0	1546,1403	$C_{86}H_{163}O_{20}P$	1.6	Ac_2PIM_1	$1C_{16}$, $3C_{18}$
1638.2	1638,1148	$C_{87}H_{163}O_{25}P$	8.1	Ac_2PIM_2	$3C_{16}$, $1C_{18}$
1652.2	1652,1305	$C_{88}H_{165}O_{25}P$	3.3	Ac_2PIM_2	$3C_{16}$, $1C_{19}$
1666.2	1666,1461	$C_{89}H_{167}O_{25}P$	16.2	Ac_2PIM_2	$2C_{16}$, $2C_{18}$
1680.2	1680,1618	$C_{90}H_{169}O_{25}P$	4.3	Ac_2PIM_2	$2C_{16}$, $1C_{18,}$ $1C_{19}$
1694.2	1694,1774	$C_{91}H_{171}O_{25}P$	5.1	Ac_2PIM_2	$2C_{16}$, $2C_{19}$
1800.2	1800,1677	$C_{93}H_{173}O_{30}P$	1.7	Ac_2PIM_3	$4C_{16}$
1828,3	1828,1990	$C_{95}H_{177}O_{30}P$	4.1	Ac_2PIM_3	$2C_{16}$, $2C_{18}$
2062.1	2062,1121	$C_{96}H_{175}O_{44}P$	1.2	Ac_1PIM_6	$2C_{16}$, $1C_{19}$
2342.4	2342,3887	$C_{115}H_{211}O_{45}P$	0.9	Ac_2PIM_6	$2C_{16}$, $2C_{19}$

[a] the intensity of PI was not taken into account

Supplementary Table 5. List of the [M-H]$^-$ ions of the Ac_4SGL and corresponding molecular species detected by MALDI-TOF mass spectrometry in Ch S$^+$/C$^+$ pool.

Peak assignment was performed according to previous studies[4,5]. SL-I and SL-II refer to the nomenclature introduced by Goren *et al.*[6]. SL-I are acylated by 1 palmitic or 1 stearic acid ($C_{16/18}$), 2 hydroxyphthioceranoïc acid (HPA) and 1 phthioceranoïc acid (PA); SL-II are acylated by 1 palmitic or 1 stearic acid ($C_{16/18}$), and 3 HPA (See Fig. 3). The last column indicates the cumulated number of carbon atoms in the 3 HPA or PA chains; the first and second number correspond to molecules acylated by the C_{16} and the C_{18}, respectively.
Ac_4SGL from Mtb_broth show a massif of ions centered at *m/z* from 2459 to 2543[4,5] (see below in blue), whereas Ac_4SGL in EBCs show a massif of ions centered at *m/z* from 2739 to 2823 (see below in red; Fig. 3). n.d., not determined.

Measured *m/z* of [M-H]$^-$	Calculated *m/z* of [M-H]$^-$	Molecular formula of M	Relative Intensity (%)	Species SL-I : 2HPA, 1PA SL-II : 3 HPA	Total chain length of the 3 HPA/PA (carbon atom number)
2471,2	2471,1636	$C_{152} H_{293} O_{20} S$	n.d.	SL-I	124 / 122
2473,2	2473,1429	$C_{151} H_{291} O_{21} S$	1,0	SL-II	123 / 121
2485,2	2485,1793	$C_{153} H_{295} O_{20} S$	0,7	SL-I	125 / 123
2487,2	2487,1586	$C_{152} H_{293} O_{21} S$	0,8	SL-II	124 / 122
2499,2	2499,1949	$C_{154} H_{297} O_{20} S$	1,0	SL-I	126 / 124
2501,2	2501,1742	$C_{153} H_{295} O_{21} S$	1,0	SL-II	125 / 123
2513,2	2513,2106	$C_{155} H_{299} O_{20} S$	0,8	SL-I	127 / 125
2515,2	2515,1899	$C_{154} H_{297} O_{21} S$	1,0	SL-II	126 / 124
2527,2	2527,2262	$C_{156} H_{301} O_{20} S$	0,9	SL-I	128 / 126
2529,2	2529,2055	$C_{155} H_{299} O_{21} S$	0,9	SL-II	127 / 125
2541,2	2541,2419	$C_{157} H_{303} O_{20} S$	1,2	SL-I	129 / 127
2543,2	2543,2212	$C_{156} H_{301} O_{21} S$	0,8	SL-II	128 / 126
2555,2	2555,2575	$C_{158} H_{305} O_{20} S$	0,9	SL-I	130 / 128
2557,2	2557,2368	$C_{157} H_{303} O_{21} S$	1,1	SL-II	129 / 127
2569,2	2569,2732	$C_{159} H_{307} O_{20} S$	1,3	SL-I	131 / 129
2571,2	2571,2525	$C_{158} H_{305} O_{21} S$	1,7	SL-II	130 / 128
2583,2	2583,2888	$C_{160} H_{309} O_{20} S$	0,9	SL-I	132 / 130
2585,2	2585,2681	$C_{159} H_{307} O_{21} S$	1,8	SL-II	131 / 129
2597,2	2597,3045	$C_{161} H_{311} O_{20} S$	0,7	SL-I	133 / 131
2599,2	2599,2838	$C_{160} H_{309} O_{21} S$	2,1	SL-II	132 / 130
2611,3	2611,3201	$C_{162} H_{313} O_{20} S$	0,9	SL-I	134 / 132
2613,3	2613,2994	$C_{161} H_{311} O_{21} S$	1,5	SL-II	133 / 131
2625,3	2625,3358	$C_{163} H_{315} O_{20} S$	1,3	SL-I	135 / 133
2627,3	2627,3151	$C_{162} H_{313} O_{21} S$	1,5	SL-II	134 / 132
2639,3	2639,3514	$C_{164} H_{317} O_{20} S$	1,2	SL-I	136 / 134
2641,3	2641,3307	$C_{163} H_{315} O_{21} S$	1,9	SL-II	135 / 133
2653,3	2653,3671	$C_{165} H_{319} O_{20} S$	1,4	SL-I	137 / 135
2655,3	2655,3464	$C_{164} H_{317} O_{21} S$	2,0	SL-II	136 / 134
2667,3	2667,3827	$C_{166} H_{321} O_{20} S$	1,2	SL-I	138 / 136
2669,3	2669,3620	$C_{165} H_{319} O_{21} S$	2,2	SL-II	137 / 135
2681,3	2681,3984	$C_{167} H_{323} O_{20} S$	1,6	SL-I	139 / 137
2683,3	2683,3777	$C_{166} H_{321} O_{21} S$	1,8	SL-II	138 / 136
2695,3	2695,4140	$C_{168} H_{325} O_{20} S$	1,5	SL-I	140 / 138
2697,3	2697,3933	$C_{167} H_{323} O_{21} S$	2,7	SL-II	139 / 137
2709,3	2709,4297	$C_{169} H_{327} O_{20} S$	2,0	SL-I	141 / 139
2711,3	2711,4090	$C_{168} H_{325} O_{21} S$	2,9	SL-II	140 / 138
2723,3	2723,4453	$C_{170} H_{329} O_{20} S$	1,5	SL-I	142 / 140

2725,3	2725,4246	$C_{169} H_{327} O_{21} S$	2,6	SL-II	141 / 139
2737,3	2737,4610	$C_{171} H_{331} O_{20} S$	1,7	SL-I	143 / 141
2739,3	2739,4403	$C_{170} H_{329} O_{21} S$	2,9	SL-II	142 / 140
2751,3	2751,4766	$C_{172} H_{333} O_{20} S$	2,0	SL-I	144 / 142
2753,3	2753,4559	$C_{171} H_{331} O_{21} S$	3,3	SL-II	143 / 141
2765,3	2765,4923	$C_{173} H_{335} O_{20} S$	2,3	SL-I	145 / 143
2767,3	2767,4716	$C_{172} H_{333} O_{21} S$	2,8	SL-II	144 / 142
2779,4	2779,5079	$C_{174} H_{337} O_{20} S$	1,8	SL-I	146 / 144
2781,4	2781,4872	$C_{173} H_{335} O_{21} S$	3,0	SL-II	145 / 143
2793,4	2793,5236	$C_{175} H_{339} O_{20} S$	1,3	SL-I	147 / 145
2795,4	2795,5029	$C_{174} H_{337} O_{21} S$	2,9	SL-II	146 / 144
2807,4	2807,5392	$C_{176} H_{341} O_{20} S$	1,6	SL-I	148 / 146
2809,4	2809,5185	$C_{175} H_{339} O_{21} S$	3,3	SL-II	147 / 145
2821,4	2821,5549	$C_{177} H_{343} O_{20} S$	1,3	SL-I	149 / 147
2823,4	2823,5342	$C_{176} H_{341} O_{21} S$	3,0	SL-II	148 / 146
2835,4	2835,5705	$C_{178} H_{345} O_{20} S$	1,6	SL-I	150 / 148
2837,4	2837,5498	$C_{177} H_{343} O_{21} S$	2,6	SL-II	149 / 147
2849,4	2849,5862	$C_{179} H_{347} O_{20} S$	1,2	SL-I	151 / 149
2851,4	2851,5654	$C_{178} H_{345} O_{21} S$	1,7	SL-II	150 / 148
2863,4	2863,6018	$C_{180} H_{349} O_{20} S$	1,0	SL-I	152 / 150
2865,4	2865,5811	$C_{179} H_{347} O_{21} S$	1,6	SL-II	151 / 149
2877,4	2877,6175	$C_{181} H_{351} O_{20} S$	0,8	SL-I	153 / 151
2879,4	2879,5968	$C_{180} H_{349} O_{21} S$	1,0	SL-II	152 / 150
2891,4	2891,6331	$C_{182} H_{353} O_{20} S$	0,6	SL-I	154 / 152
2893,4	2893,6124	$C_{181} H_{351} O_{21} S$	1,2	SL-II	153 / 151
2905,5	2905,6488	$C_{183} H_{355} O_{20} S$	0,4	SL-I	155 / 153
2907,4	2907,6281	$C_{182} H_{353} O_{21} S$	0,6	SL-II	154 / 152
2919,4	2919,6644	$C_{184} H_{357} O_{20} S$	n.d.	SL-I	156 / 154
2921,5	2921,6437	$C_{183} H_{355} O_{21} S$	n.d.	SL-II	155 / 153

Supplementary Table 6. List of the $[M+NH_4]^+$ ions of the PDIM and corresponding major molecular species detected by ESI-QTOF mass spectrometry in Ch S^+/C^+ pool.

Peak assignment was performed according to previous studies[7,8]. In PDIMA, 2 mycocerosic acids (MCA) esterify a phthiocerol chain (Fig. 3). The last column indicates the cumulated number of carbon atoms in the 2 MCA chains.

PDIM from Mtb_broth show major forms from *m/z* 1371 to 1427 (see below in blue), depending of the *M. tuberculosis* strain, H37Rv, Erdman or Mt103[7-9], whereas PDIM in EBC show increased abundance of the forms at *m/z* 1469 and 1497 (see below in red; Fig. 3).

Measured *m/z* of $[M+NH4]^+$ adducts	Calculated *m/z* of $[M+NH4]^+$ adducts	Absolute Error (ppm)	Molecular formula of M	Relative Intensity (%)	Total chain length of the 2 MCA (carbon atom number)
1343,3804	1343,3856	3,87	$C_{89}H_{176}O_5$	2,4	53-56
1357,3953	1357,4013	4,42	$C_{90}H_{178}O_5$	2,6	54-57
1371,4132	1371,4169	2,70	$C_{91}H_{180}O_5$	4,0	55-58
1385,4377	1385,4326	3,68	$C_{92}H_{182}O_5$	9,7	56-59
1399,4492	1399,4482	0,71	$C_{93}H_{184}O_5$	5,7	57-60
1413,4642	1413,4639	0,21	$C_{94}H_{186}O_5$	8,9	58-61
1427,4838	1427,4795	3,01	$C_{95}H_{188}O_5$	10,6	59-62
1441,4980	1441,4952	1,94	$C_{96}H_{190}O_5$	10,0	60-63
1455,5133	1455,5108	1,72	$C_{97}H_{192}O_5$	10,6	61-64
1469,5308	1469,5265	2,93	$C_{98}H_{194}O_5$	18,8	62-65
1483,5408	1483,5421	0,88	$C_{99}H_{196}O_5$	5,1	63-66
1497,5616	1497,5578	2,54	$C_{100}H_{198}O_5$	9,4	64-67
1511,5732	1511,5734	0,13	$C_{101}H_{200}O_5$	1,8	65-68
1525,5882	1525,5891	0,59	$C_{102}H_{202}O_5$	0,5	66-69

Supplementary Table 7. List of the [M-H]⁻ ions of the mycolic acids and corresponding major molecular species detected by ESI-QTOF mass spectrometry in Ch S^+/C^+ pool. Peak assignment was performed according to previous studies[10].
The distribution of the free mycolic acids molecular species in EBC (Fig. 3) is very similar to that observed for mycolic acid esters in Mtb_broth[10-12].

Measured *m/z* of $[M-H]^-$	Calculated *m/z* of $[M-H]^-$	Absolute Error (ppm)	Molecular formula of M	Relative Intensity (%)	Species	Total carbon number
1108,1406	1108,1356	4,51	C_{76} H1$_{48}$ O_3	2.4	α-	76
1122,1537	1122,1512	2,23	$C_{77}H_{150}O_3$	2.1	α-	77
1136,1716	1136,1669	4,14	$C_{78}H_{152}O_3$	27.7	α-	78
1150,1857	1150,1825	2,78	$C_{79}H_{154}O_3$	3.6	α-	79
1164,2111	1164,1982	11,08	$C_{80}H_{156}O_3$	10.5	α-	80
1178,2152	1178,2138	1,19	$C_{81}H_{158}O_3$	0.5	α-	81
1192,2289	1192,2295	0,50	$C_{82}H_{160}O_3$	0.2	α-	82
1224,2585	1224,2557	2,29	$C_{83}H_{164}O_4$	2.0	Methoxy-	83
1238,2712	1238,2713	0,08	$C_{84}H_{166}O_4$	1.3	Methoxy-	84
1252,2955	1252,2870	6,79	$C_{85}H_{168}O_4$	15.9	Methoxy-	85
1266,3046	1266,3026	1,58	$C_{86}H_{170}O_4$	1.9	Methoxy-	86
1280,3279	1280,3183	7,50	$C_{87}H_{172}O_4$	18.2	Methoxy-	87
1294,3385	1294,3339	3,55	$C_{88}H_{174}O_4$	2.6	Methoxy-	88
1308,3542	1308,3496	3,52	$C_{89}H_{176}O_4$	5.6	Methoxy-	89
1322,3693	1322,3652	3,10	$C_{90}H_{178}O_4$	2.8	Methoxy-	90
1336,3824	1336,3809	1,12	$C_{91}H_{180}O_4$	0.9	Methoxy-	91
1350,3977	1350,3965	0,89	$C_{92}H_{182}O_4$	2.0	Methoxy-	92

Supplementary Table 8: Number of proteins detected by proteomic analysis in individual EBCs.

Number of proteins detected	Ad S^+	Ad healthy	Ad pneumo	Ch S^+/C^+	Ch healthy
M. tuberculosis					
**Minimal**	147	0	0	14	0
**Maximal**	1288	0	0	17	0
**Cumulated**	1432	0	0	23	0
**Human**					
**Minimal**	161	248	232	121	204
**Maximal**	189	282	275	203	274
**Cumulated**	331	348	328	227	365

Supplementary Data 1: List of Mtb proteins detected by proteomic analysis in individual EBCs.

Supplementary Data 2: Abundance of proteins detected by proteomic analysis in individual EBCs.

Proteins highlighted in blue were previously described to be released in extracellular vesicles[13-16]. The citing reference(s) are indicated for each protein.

REFERENCES

1. Gilleron, M., *et al.* Acylation state of the phosphatidylinositol mannosides from Mycobacterium bovis bacillus Calmette Guerin and ability to induce granuloma and recruit natural killer T cells. *J Biol Chem* **276**, 34896-34904 (2001).
2. Gilleron, M., Quesniaux, V.F. & Puzo, G. Acylation state of the phosphatidylinositol hexamannosides from Mycobacterium bovis bacillus Calmette Guerin and mycobacterium tuberculosis H37Rv and its implication in Toll-like receptor response. *J Biol Chem* **278**, 29880-29889 (2003).
3. Gilleron, M., Lindner, B. & Puzo, G. MS/MS approach for characterization of the fatty acid distribution on mycobacterial phosphatidyl-myo-inositol mannosides. *Anal Chem* **78**, 8543-8548 (2006).
4. Layre, E., *et al.* Deciphering sulfoglycolipids of Mycobacterium tuberculosis. *J Lipid Res* **52**, 1098-1110 (2011).
5. Rhoades, E.R., Streeter, C., Turk, J. & Hsu, F.F. Characterization of sulfolipids of Mycobacterium tuberculosis H37Rv by multiple-stage linear ion-trap high-resolution mass spectrometry with electrospray ionization reveals that the family of sulfolipid II predominates. *Biochemistry* **50**, 9135-9147 (2011).
6. Goren, M.B., Brokl, O., Das, B.C. & Lederer, E. Sulfolipid I of Mycobacterium tuberculosis, strain H37RV. Nature of the acyl substituents. *Biochemistry* **10**, 72-81 (1971).
7. Camacho, L.R., *et al.* Analysis of the phthiocerol dimycocerosate locus of Mycobacterium tuberculosis. Evidence that this lipid is involved in the cell wall permeability barrier. *J Biol Chem* **276**, 19845-19854 (2001).
8. Jain, M., *et al.* Lipidomics reveals control of Mycobacterium tuberculosis virulence lipids via metabolic coupling. *Proc Natl Acad Sci U S A* **104**, 5133-5138 (2007).
9. Augenstreich, J., *et al.* The conical shape of DIM lipids promotes Mycobacterium tuberculosis infection of macrophages. *Proc Natl Acad Sci U S A* **116**, 25649-25658 (2019).
10. Laval, F., Laneelle, M.A., Deon, C., Monsarrat, B. & Daffe, M. Accurate molecular mass determination of mycolic acids by MALDI-TOF mass spectrometry. *Anal Chem* **73**, 4537-4544 (2001).
11. Minnikin, D.E. & Brennan, P.J. Lipids of Clinically Significant Mycobacteria. in *Health Consequences of Microbial Interactions with Hydrocarbons, Oils, and Lipids* (ed. Goldfine, H.) 1-76 (Springer Nature Switzerland AG 2020, 2020).
12. Marrakchi, H., Laneelle, M.A. & Daffe, M. Mycolic acids: structures, biosynthesis, and beyond. *Chem Biol* **21**, 67-85 (2014).
13. Giri, P.K., Kruh, N.A., Dobos, K.M. & Schorey, J.S. Proteomic analysis identifies highly antigenic proteins in exosomes from M. tuberculosis-infected and culture filtrate protein-treated macrophages. *Proteomics* **10**, 3190-3202 (2010).
14. Lee, J., *et al.* Proteomic analysis of extracellular vesicles derived from Mycobacterium tuberculosis. *Proteomics* **15**, 3331-3337 (2015).
15. Palacios, A., Gupta, S., Rodriguez, G.M. & Prados-Rosales, R. Extracellular vesicles in the context of Mycobacterium tuberculosis infection. *Mol Immunol* **133**, 175-181 (2021).
16. Prados-Rosales, R., *et al.* Mycobacteria release active membrane vesicles that modulate immune responses in a TLR2-dependent manner in mice. *J Clin Invest* **121**, 1471-1483 (2011).

Uncropped scans of Supplementary Fig. 2.

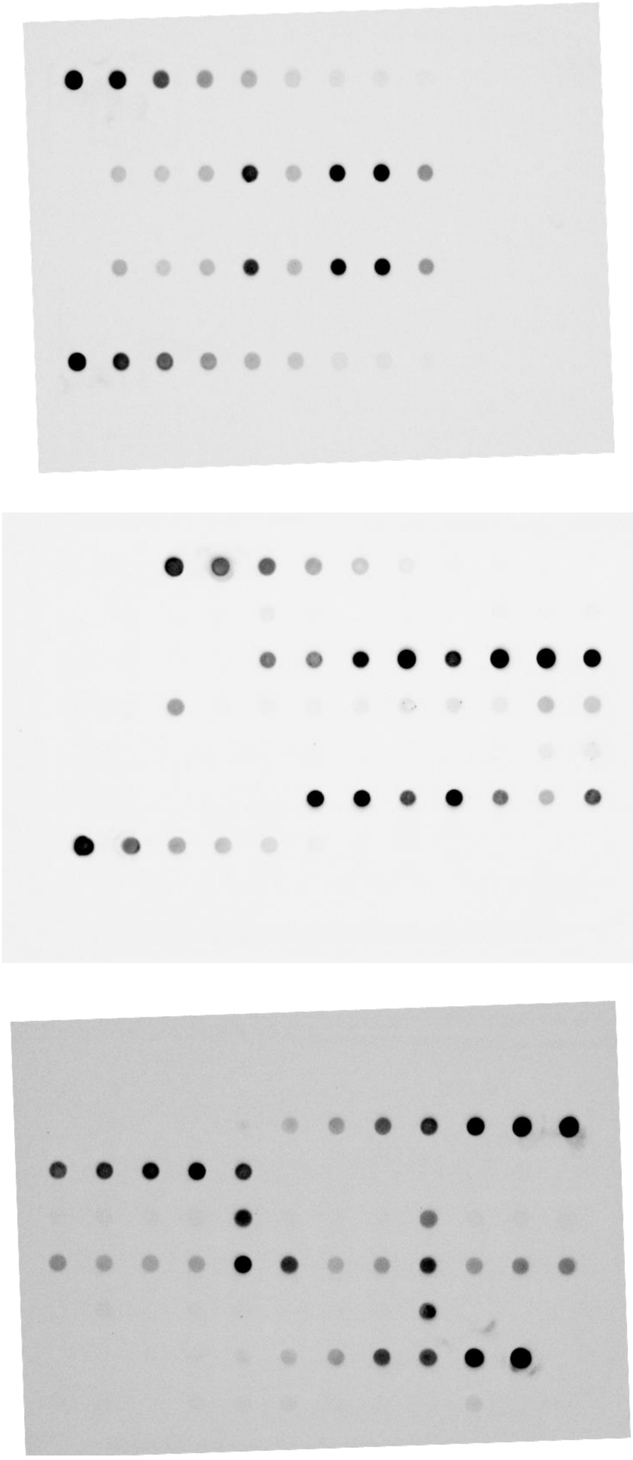

Uncropped scan of Supplementary Fig. 7.

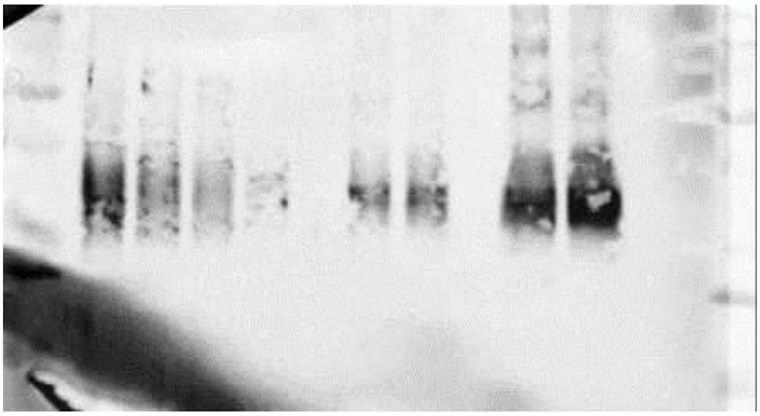

Supplement: Supplementary file 1 — Supplementary information [file 41467_2022_35453_MOESM1_ESM.pdf]
