# Supplementary material for: A Mycobacterium tuberculosis fingerprint in human breath allows tuberculosis detection
Source: Nat Commun. 2022 Dec 14;13:7751. doi: 10.1038/s41467-022-35453-5 (PMC9751131; doi:10.1038/s41467-022-35453-5)
Supplement: Supplementary file 2 — Description to Additional Supplementary Information [file 41467_2022_35453_MOESM2_ESM.pdf]

Description of Additional Supplementary Files

Supplementary Data 1: List of Mtb proteins detected by proteomic analysis in individual EBCs.

Supplementary Data 2: Abundance of proteins detected by proteomic analysis in individual EBCs.
