# Supplementary material for: A Mycobacterium tuberculosis fingerprint in human breath allows tuberculosis detection
Source: Nat Commun. 2022 Dec 14;13:7751. doi: 10.1038/s41467-022-35453-5 (PMC9751131; doi:10.1038/s41467-022-35453-5)
Supplement: Supplementary file 5 — Reporting Summary [file 41467_2022_35453_MOESM5_ESM.pdf]

Corresponding author(s): Jérôme Nigou

Last updated by author(s): Nov 28, 2022

Reporting Summary

Nature Research wishes to improve the reproducibility of the work that we publish. This form provides structure for consistency and transparency in reporting. For further information on Nature Research policies, see our Editorial Policies and the Editorial Policy Checklist.

Statistics

For all statistical analyses, confirm that the following items are present in the figure legend, table legend, main text, or Methods section.

n/a	Confirmed	
☐	☒	The exact sample size (*n*) for each experimental group/condition, given as a discrete number and unit of measurement
☐	☒	A statement on whether measurements were taken from distinct samples or whether the same sample was measured repeatedly
☐	☒	The statistical test(s) used AND whether they are one- or two-sided<br>*Only common tests should be described solely by name; describe more complex techniques in the Methods section.*
☒	☐	A description of all covariates tested
☒	☐	A description of any assumptions or corrections, such as tests of normality and adjustment for multiple comparisons
☐	☒	A full description of the statistical parameters including central tendency (e.g. means) or other basic estimates (e.g. regression coefficient) AND variation (e.g. standard deviation) or associated estimates of uncertainty (e.g. confidence intervals)
☐	☒	For null hypothesis testing, the test statistic (e.g. *F*, *t*, *r*) with confidence intervals, effect sizes, degrees of freedom and *P* value noted<br>*Give P values as exact values whenever suitable.*
☒	☐	For Bayesian analysis, information on the choice of priors and Markov chain Monte Carlo settings
☒	☐	For hierarchical and complex designs, identification of the appropriate level for tests and full reporting of outcomes
☒	☐	Estimates of effect sizes (e.g. Cohen's *d*, Pearson's *r*), indicating how they were calculated

Our web collection on statistics for biologists contains articles on many of the points above.

Software and code

Policy information about availability of computer code

Data collection	Image Lab (version 6.0.1 build 34); MassLynx (version 4.1); Xcalibur (version 4.3.73.11); Topspin (version 3.5 PL7)
Data analysis	MassLynx (version 4.2); Mascot (version 2.7.0); Proline (version 2.1.2); Prism 5.0 (GraphPad Software); R (version 4.2.1); caret package (version 6.0-93); tidyverse (version 1.3.2)

For manuscripts utilizing custom algorithms or software that are central to the research but not yet described in published literature, software must be made available to editors and reviewers. We strongly encourage code deposition in a community repository (e.g. GitHub). See the Nature Research guidelines for submitting code & software for further information.

Data

Policy information about availability of data

All manuscripts must include a data availability statement. This statement should provide the following information, where applicable:

- Accession codes, unique identifiers, or web links for publicly available datasets
- A list of figures that have associated raw data
- A description of any restrictions on data availability

Raw data are provided as a Source Data file. The mass spectrometry proteomics data were searched against MTB_H37Rv_native and Human entries of the UniProtKB protein database (release Swiss-Prot+TrEMBL 2020_10, 4,080 entries and release Swiss-Prot 2020_10, 20,385 entries, respectively), and have been deposited to the ProteomeXchange Consortium via the PRIDE partner repository with the dataset identifier PXD028477.

Field-specific reporting

Please select the one below that is the best fit for your research. If you are not sure, read the appropriate sections before making your selection.

☒ Life sciences ☐ Behavioural & social sciences ☐ Ecological, evolutionary & environmental sciences

For a reference copy of the document with all sections, see nature.com/documents/nr-reporting-summary-flat.pdf

Life sciences study design

All studies must disclose on these points even when the disclosure is negative.

Sample size	No sample size calculation was performed. Patients were included prospectively. Considering that our study is a pilot study, the number of patients (n=46) and control individuals (n=45) is in the range of similar studies in the field (Paris et al., Sci Transl. Med., 2017, 9:eaal2807; Chen et al., Sci. Rep., 2020, 10:7647).
Data exclusions	No data were excluded from the analyses.
Replication	EBC samples are unique. Values shown in graphs or tables are the mean of one to several independent measurements (technical replicates) on each individual EBC sample. Technical replicates were successful as indicated in the figure legends where it applies.
Randomization	For in vitro measurements, randomization was not relevant. Patients were included prospectively. Randomization was not relevant for the study. Patients were grouped according to their clinical status.
Blinding	Investigators were not blinded in this study because the same investigators had to perform various steps of the experiments.

Reporting for specific materials, systems and methods

We require information from authors about some types of materials, experimental systems and methods used in many studies. Here, indicate whether each material, system or method listed is relevant to your study. If you are not sure if a list item applies to your research, read the appropriate section before selecting a response.

Materials & experimental systems

n/a	Involved in the study	
☐	☒	Antibodies
☒	☐	Eukaryotic cell lines
☒	☐	Palaeontology and archaeology
☒	☐	Animals and other organisms
☐	☒	Human research participants
☒	☐	Clinical data
☒	☐	Dual use research of concern

Methods

n/a	Involved in the study	
☒	☐	ChIP-seq
☒	☐	Flow cytometry
☒	☐	MRI-based neuroimaging

Antibodies

Antibodies used	Primary antibodies used were obtained through BEI Resources, NIAID, NIH: Monoclonal Anti-Mycobacterium tuberculosis LAM, Clone CS-35 (produced in vitro; dilution of 1:200), NR-13811; Monoclonal Anti-Mycobacterium tuberculosis GroEL2 (Gene Rv0440), Clone CS-44 (produced in vitro; dilution of 1:1000), NR-13813; Monoclonal Anti-Mycobacterium tuberculosis HspX (Gene Rv2031c), Clone IT-20 (TB68) (produced in vitro; dilution of 1:100), NR-13607; Monoclonal Anti-Mycobacterium tuberculosis KatG (Gene Rv1908c), Clone IT-57 (CDA4) (culture supernatant; dilution of 1:200), NR-13793; Monoclonal Anti-Mycobacterium tuberculosis LpqH (Gene Rv3763), Clone IT-54 (produced in vitro; dilution of 1:200), NR-13792; Monoclonal Anti-Mycobacterium tuberculosis HBHA (Gene Rv0475), Clone α-HBHA (produced in vitro; dilution of 1:50), NR-13804.<br>The secondary antibodies used were goat anti-mouse IgG3-HRP (ThermoFisher; dilution of 1:2000) or goat anti-mouse IgG2b-HRP (Invitrogen; dilution of 1:5000).
Validation	Validation of the primary antibodies can be found on the BEI website: https://www.beiresources.org/

Human research participants

Policy information about studies involving human research participants

Population characteristics	Population characteristics can be found in the manuscript Table 1 and Extended Data Table 1.
Recruitment	Adults and children patients with TB were recruited through the TB Control Program of the Secretaría de Salud de Medellín

Recruitment	and the Secretaría Seccional de Salud y Bienestar Social de Antioquia, Colombia. The diagnostic tests for TB and HIV were performed in the laboratories of health centers where the patients received primary care. Patients were included prospectively after diagnosis by the clinicians, independently of the article authors, avoiding any self-selection bias. Exclusion criteria were: patients positive for HIV, diabetes, cancer, autoimmune diseases, immunosuppressive treatment, smoking, or previous TB.<br>Control patients with Community-Acquired Pneumonia were adults. Inclusion criteria were age ≥ 18, fever (>38 °C), cough, increased respiratory rate or respiratory distress, infiltrates on chest radiographs and bacterial pneumonia confirmed in sputum. Exclusion criteria were immunodeficiency, chronic lung or heart diseases, neoplasia, hospital-acquired pneumonia or viral pneumonia. Twelve patients had pneumonia with consolidation and three without. All of them presented complicated pneumonia, including 8 bacteremias, 4 empyemas, and 3 pleural effusions.<br>Healthy control adults were individuals who did not have respiratory symptoms nor a history of tuberculosis. They were recruited by the medical and laboratory personnel from the Facultad de Medicina, Universidad de Antioquia. Healthy control children had no previous contact with TB patients nor respiratory symptoms were also recruited. The parents consented to participation of children in this study and informed the researchers that the children were BCG vaccinated.
Ethics oversight	The Ethics Committee of the Facultad de Medicina, Universidad de Antioquia, approved the study. Participants did not receive any compensation.

Note that full information on the approval of the study protocol must also be provided in the manuscript.
